# Internal Consistency Does Not Validate an LLM-Assisted Nutrition Pipeline: A Reference-Standard-Free Forensic Reliability Audit of 13,073 Free-Text Meal Records

**DOI:** 10.3390/nu18193233

**Published:** 2026-09-30

**Authors:** Jhony Fernando López Torres, Juan Gabriel Castañeda, Edwin Rivas

**Affiliations:** 1 Neptura, Libano 731040, Tolima, Colombia; 2 Facultad de Ciencias Humanas, Corporación Universitaria Minuto de Dios—UNIMINUTO, Bogotá D.C. 111021, Colombia; jucastaneda@uniminuto.edu; 3 Facultad de Ingeniería, Universidad Distrital Francisco José de Caldas, Bogotá D.C. 111711, Colombia; erivas@udistrital.edu.co

**Keywords:** diet records, nutrition assessment, reproducibility of results, artificial intelligence, data accuracy, large language models

## Abstract

Background/Objectives: Large language model (LLM) pipelines increasingly convert free-text meal descriptions into nutrient values, yet reference-standard validation is rarely feasible in production. We asked whether the controls such pipelines conventionally pass suffice and built a reference-standard-free battery any pipeline can run on its output. Methods: We audited 13,073 meals logged by 513 adults through a WhatsApp conversational agent in rural Colombia (9 February 2026 to 6 August 2026). The pipeline is a two-stage cascade of commercial LLMs from different providers: a vision model renders photographs as text, while a text model maps these to nutrients. Six pre-specified tests were applied, including test–retest ICC(1,1) over recurring strings, stratified by modality because 48.3% of records carried a photograph. Results: The pipeline passed every conventional control (Atwater-reconstructed energy r = 0.9951; median relative error 0.0000) but failed reproducibility. Across 191 recurring strings (944 meals), energy ICC point estimates were 0.7061 (95% CI 0.5419–0.8217) text only and 0.5258 (0.3778–0.6513) with photographs; none reached 0.75. Modality is a design control, not an established effect, and the coefficient depends on which inputs recur, so it is not intrinsic. Under the strictest identity available—byte-identical text, no photograph—ICC was 0.6944. Intermediate-text inspection exposed a failure invisible at the output: 1.44% of photograph-accompanied records carried a vision-stage artefact string versus 0.01% of text-only records (ICC 0.0911). Conclusions: Internal consistency does not validate an LLM-assisted nutrition pipeline. This design measures instability under recurring inputs without isolating its cause. Prospective test–retest reproducibility is a cheap, necessary acceptance criterion, and in a cascade, the intermediate representation must also be inspected.

## 1. Introduction

Conversational agents that accept a free-text meal description and return a nutrient breakdown are now embedded in consumer applications and in public health programmes. The appeal is obvious: a participant types “arepa con huevo” and the system returns energy, protein, carbohydrate, fat, fibre, and sugar without a trained interviewer, a food scale, or a composition-table lookup performed by hand.

The evidence base supporting these engines has been built almost entirely on accuracy. Published evaluations contrast a single estimate against a weighed portion or a reference composition database: GPT-4 against 114 photographed meals from a national survey [1]; three commercial models against standardised food photographs with direct weighing as the comparator [2]; ChatGPT-4 against 38 photographs of known composition [3]; a retrieval-augmented framework anchored in FNDDS and evaluated against ASA24 [4]; and three models against the consensus judgement of two human experts on 1992 food items from a Polish long-term-care cohort [5]. All are photograph-driven, all compare against an external standard, and all issue one invocation per item.

That design choice is inherited from the wider evaluation literature on clinical language models, which has concentrated on average correctness against a gold standard: performance on licensing-examination items, on clinical text summarisation against expert reference summaries, and on expert-level question answering [6,7,8]. Where that literature has looked beyond mean accuracy, it has found the failure modes to be systematic rather than random: models that answer well in aggregate can follow incorrect reasoning paths and are sensitive to how the case is presented [9]. Proposals for human evaluation frameworks and for regulatory oversight of deployed models both start from the premise that a single accuracy figure is an insufficient acceptance criterion [10,11], and reporting guidance for prediction models built on these systems now asks explicitly how the model version and its inputs were fixed [12].

This design leaves a question unasked. An engine can be accurate on average and still return a different number every time it is shown the same input. Accuracy and reproducibility are distinct instrument properties—a distinction formalised for health measurement instruments in the COSMIN taxonomy [13]—and only one of them can be measured after deployment without a reference standard. A biased but reproducible instrument is calibratable; an irreproducible one is not, because there is no stable quantity to calibrate.

Outside quantitative nutrition, the design of resubmitting an identical input to the same model is established. Repeating each question five times against orthopedic guidelines produced Fleiss kappa values ranging from −0.002 to 0.984 [14]; nutritional advice requested on 3 consecutive days replicated in 50–86% of cases [15]; consistency has been measured on registered-dietitian examination items [16]; and five independent sessions per clinical vignette yielded internal consistency as low as 0.60 for inpatient management decisions [17,18]. In every case, the output is categorical or ordinal, never the continuous nutrient values a diet record requires.

Parallel literature gives us the language for the consequences. Dietary measurement error attenuates diet–disease associations toward the null, and correcting for it requires knowing the instrument’s reliability [19,20]; repeat measurements are the standard route to estimating that dilution at scale [21,22]. This body of work, however, models error in the human informant. The error we describe here originates in the algorithmic estimator, is independent of the informant, and—unlike human error—can be measured at essentially zero cost by resending text.

Finally, data-integrity screening offers a ready instrument for a different question: whether the returned numbers are as granular as they claim. Digit-distribution analysis has been used to detect fabrication in clinical trials [23], to screen for misconduct via Benford’s law [24], to document terminal-digit preference as a measurement bias in endoscopy [25], and to assess anthropometric data quality in nutrition surveys [26]. To our knowledge, it has not been turned on the output of a generative estimator.

Our specific aims were: (i) to formalise a six-test forensic battery portable to any deployed pipeline; (ii) to estimate test–retest reliability on inputs that recurred in the operational record, a design whose limits relative to true resubmission we quantify in Section 3.4.3; and (iii) to quantify what the resulting noise costs a nutrition cohort in statistical power.

## 2. Methods

### 2.1. Design and Reporting Guideline

This is a retrospective reliability and agreement study of a system in production (a deployed-system reliability audit). It is explicitly not a criterion-validity study: no reference standard existed (Section 4.3). The primary observation unit is the meal record; the unit of the reliability substudy is the recurring identical text string; the unit of the attenuation analysis is the participant.

We report according to GRRAS [27]. Reporting guidance specific to studies of large language models in health has since appeared [28]; it targets predictive and generative clinical tasks rather than instrument reliability, so GRRAS remains the primary framework here, and TRIPOD-LLM is used as a secondary check on the description of the model and its deployment. GRRAS was designed for studies of agreement between human observers or instruments. Here, the “observer” is the estimation engine and the “replicates” are separate invocations on inputs that recurred in the operational record. They are not resubmissions performed by the investigators, and Section 3.4.3 quantifies how far they fall short of that standard. This adaptation is stated explicitly, and the item-by-item checklist accompanies this manuscript as Supplementary Materials.

### 2.2. Setting and Data Source

The parent programme holds ethics approval from the Research Ethics Committee (Comité de Ética en Investigación) of the Universidad Distrital Francisco José de Caldas, Bogotá, Colombia (approval code: programme-level endorsement (call 949 of 2024); approval date: 17 June 2024 (see Institutional Review Board Statement); the present analysis is a secondary study of records that programme generated in routine operation.

Adult registrants in the BIOMARCADORES-111598 metabolic-risk screening programme logged meals spontaneously through a WhatsApp conversational agent—a delivery channel whose reach and limitations in health care are documented in scoping reviews of conversational agents and in feasibility studies of chatbots for nutrition and physical activity [29,30], and reviewed more broadly elsewhere [31]—in the rural municipalities of Honda (Tolima) and Guacarí (Valle del Cauca), Colombia. Each entry consists of a free-text description in Spanish, optionally accompanied by a photograph, which the pipeline converts into six numeric fields: energy (kcal), protein, carbohydrate, fat, fibre, and sugar (g). The pipeline is a two-stage cascade of commercial large language models from different providers: a vision-capable model first renders any photograph as a Spanish text description, and a second, text-only model maps the resulting description—whether written by the participant or by the first stage—to the six numeric fields. The model families are known to the investigators, but the prompts, sampling temperature, exact model checkpoints, and any anchoring to a food-composition database are set by the providers, are revised without notice, and can be neither inspected nor pinned by the investigators. What is audited here is therefore the behaviour of the deployed cascade as a whole, at the checkpoints in force during the observation window.

The six fields are returned together by that single call; energy is not computed downstream from the macronutrients. This matters for interpreting T5 (Section 3.1): the near-perfect Atwater agreement is not imposed by an arithmetic constraint in the pipeline, so it is informative about the model’s internal coherence—but it is a coherence check, not an accuracy check, and a model that returned mutually consistent yet uniformly wrong values would pass it. What T5 excludes is a pipeline whose energy and macronutrient fields are generated independently of each other; what it cannot address is whether either is correct.

Two distinct uses of language models must not be conflated. The cascade described in this subsection is the object of study: it generated the 13,073 nutrient records under audit, and the authors had no control over it. The assistants named in the Use of Artificial Intelligence statement are authoring tools used by the authors and played no part in producing the audited records or any reported result. Neither set of tools was involved in the other’s role.

### 2.3. Corpus and Exclusions

The audit corpus was frozen on 10 August 2026 and restricted to the declared window 9 February 2026 to 6 August 2026. Three exclusions were applied and are the only ones:

1. Test accounts. Eight hundred and sixty-eight records (836 within the window) belonging to 22 registrants flagged es_prueba in the patient registry were removed. The flag is itself missing for 158 registrants, so residual test contamination cannot be fully excluded (Section 4.3). A registrant is defined throughout by the primary registration key of the patient registry, which is populated for 49.6% of records and appears in the shared corpus as the pseudonymised registrant_id (Data Availability Statement). The registry offers two alternative keys, and counting by them instead yields 638 registrants (100% coverage) and 255 (19.6% coverage) rather than 513. Registrant counts are therefore reported as a sensitivity analysis (Table 1), and no inference in this paper depends on the participant count because the analytical unit is the meal record and the repeated string. Only the primary key was retained in the shared corpus, as a pseudonym: the two alternative keys were internal registry identifiers and were removed for data protection, so the 513 figure is reproducible from the Supplementary File, while the 638 and 255 comparators are not.
2. Re-ingestions. One hundred and twenty-five records duplicated an existing patient + date + normalised -string + value combination and were collapsed to the first occurrence. Inspection showed these to be duplicate ingestions of a single logging event—11 groups were ingested less than 60 s apart—rather than independent invocations. Retaining them inflates apparent agreement. Six of the 125 belonged to test accounts already removed by rule 1, so this rule removes 119 further records.
3. A single record with all six nutrient fields null. This record falls outside the declared window and is therefore already excluded by the window restriction; the rule is stated for completeness and removes no additional record.

The cascade is therefore 14,028 in-window records—836 test—119 re-ingestions = 13,073 meals from 513 registrants.

### 2.4. The Forensic Battery

Six tests were specified, with thresholds fixed from the digit-preference literature and conventional reliability cut points before the results of this corpus were examined (Table 2).

Their evidential status differs, and we separate it here. T1–T3 are screening tools for numerical granularity and rounding. They detect the coarseness of the output grid and cannot establish mechanism, as our own specificity simulation demonstrates by flagging legitimate non-generative processes at the same threshold (Section 3.7). T5 is a check of internal algebraic coherence, not of accuracy. Only T4 speaks to reproducibility, and it is the only test in the battery whose failure has a direct consequence for use.

### 2.5. Input Modality—A Pre-Specified Stratification

Because 48.3% of records carry an attached photograph, a repeated text string does not by itself guarantee an identical input: the engine may have received text alone on one occasion and text plus an image on another. Of the 191 repeated strings, 86 mixed both kinds of submission (45%; Supplementary Table S11). Note that the two modality strata are defined at the level of the replicate, not the string: a mixed string contributes to the text-only stratum if it has three or more text replicates and to the photograph stratum if it has three or more photograph replicates, so 28 of the 85 text-stratum strings and 34 of the 82 photograph-stratum strings are drawn from mixed strings. We therefore stratified T4 by modality before interpreting any coefficient, reporting three estimates: text-only replicates, photograph-accompanied replicates, and the pooled figure. The pooled estimate is reported for completeness but is not interpretable as a single reliability coefficient because it confounds two input conditions.

To rule out the possibility that the modality contrast merely reflects less informative text accompanying photographs, we repeated the comparison after excluding generic placeholder strings (“comida”, “comida variada”, “comida analizada”, “almuerzo”, “desayuno”, “cena”, “plato”).

### 2.6. ICC(1,1) and Interval Estimation

With a strings, N observations, and k_i_ replicates per string, ICC = (MSB − MSW)/(MSB + (k_0_ − 1)·MSW), where k_0_ = (N − Σk^2^_i_/N)/(a − 1). Confidence intervals were obtained by bootstrap resampling of strings (not of rows), 1000 replicates, seed 20260807 [35]. Interpretive cut points follow the conventional bands: <0.50 poor; 0.50–0.75 moderate; 0.75–0.90 good; >0.90 excellent [36]. The difference between modality strata was tested by bootstrapping the two ICCs independently 2000 times and taking the distribution of their difference.

Meal records are clustered in two ways—within strings and within registrants—and a string-level bootstrap addresses only the first. The two are crossed rather than nested: 41 of the 75 text-only strings recur across more than one registrant, so registrants cannot be resampled as clusters containing whole strings. We attempted a registrant-level cluster bootstrap and report it as a failed approach rather than omitting it: resampling registrants with replacement duplicates a registrant’s records inside the same string, creating exact replicates that deflate the within-string mean square by construction and push the coefficient upward until the point estimate falls outside its own interval—an impossible result that identifies the method, not the data, as the problem. Instead, we address the clustering directly by retaining one record per registrant per string and recomputing (Section 3.4.4). That analysis answers the substantive question behind the objection—whether agreement is inflated by the same person logging the same phrase repeatedly—without a resampling scheme the data structure does not support.

On the status of the 0.75 threshold. It is a convention, not a universal criterion, and we use it for one reason: it had to be fixed before the results were seen, and this is the value the reliability literature most commonly cites for the moderate/good boundary. It carries no claim that 0.75 is the level at which a nutrition estimate becomes fit for use—the defensible threshold depends on the intended inference, and for an exposure entering a correlation, the relevant quantity is the attenuation the reliability implies (Section 2.7), not the band it falls in. A pipeline at 0.80 would clear our threshold and still attenuate a true correlation by 11%. What the threshold provides is a pre-specified, externally sourced decision rule, so that the verdict does not depend on a cut point chosen after seeing the coefficient. Readers who prefer a different value can apply it: every point estimate and interval is reported, and the text-only interval straddles 0.75, so “below the threshold” is not established for that stratum at the 5% level—a distinction we keep throughout, since “not established above” and “established below” are different statements.

### 2.7. Variance Decomposition, Attenuation, and Power

Within-string mean square (MSW) estimates the variance of estimator noise at the meal level (σ^2^_e_). For an exposure measured with reliability λ, an observed regression coefficient is attenuated as β_obs = λ·β_true and a correlation as √λ. When the analytical exposure is the mean of k meals per participant, the relevant reliability is

λ_person = (Var_observed_between_persons − σ^2^_e_/k^−^)/Var_observed_between_persons,

with k^−^ the harmonic mean of meals per participant. The sample size for 80% power was computed via Fisher’s z transformation, *n* = ((z_{1 − α/2} + z_{1 − β})/z_r)^2^ + 3, the same closed form implemented in standard power software [37].

### 2.8. Specificity Analysis

To bound what T1 can and cannot establish, five non-generative number-producing mechanisms were simulated (*n* = 13,073 each, seed 11) and passed through the same flagging threshold: an exact composition-table lookup without rounding; a regression output rounded to integer; human self-report with digit heaping; a composition table displayed rounded to 5 kcal; and a packaged-food database rounded to 10 kcal.

### 2.9. Attenuation Subcohort

The bridge subcohort was rebuilt from the source registry with an explicit cascade: 1060 registry rows → 953 unique non-test registrants → 857 women (after normalising ten raw spellings of the sex field to two categories) → 116 with a laboratory fasting glucose → 116 within a plausible 60–400 mg/dL range → 70 with at least five logged meals → 62 after excluding personal diabetes diagnosis. The last exclusion required separating personal diagnosis from family history, which the registry stores in a single field; 34 rows recording only family history were retained rather than excluded.

### 2.10. Software and Use of Generative AI

Python 3.11 (pandas, numpy, scipy.stats, matplotlib). All tests ran on the frozen extract with no transformation beyond the text normalisation declared in Section 2.4. The frozen corpus, all result tables, and the figure-generating code accompany this submission.

Parts of the analysis code were drafted and debugged with the assistance of general-purpose generative AI assistants, named individually in the Use of Artificial Intelligence statement, which also records the period of use, and the language-editing use made of the same tools. Every code fragment produced was read, tested, and verified by the authors against the frozen extract before use, and the complete code is deposited as Code S1 so that any reader can re-run the analysis and confirm each reported value independently of how the code was drafted. Generative AI was not used to select or specify any analysis, to compute or generate any reported value, to produce any figure or table, to retrieve or format references, or to write any part of the Results or Discussion.

We state this in the Methods rather than only in a declaration because it bears on the argument. This manuscript reports that a deployed generative estimator does not reproduce its own numbers; the provenance of our own numbers is therefore part of what a reader is entitled to scrutinise. Every value reported here derives from deterministic computation on the frozen extract—the same input yields the same output on re-execution, which is precisely the property the audited engine lacks—and the trail from raw record to published figure is reproducible in full from the Supplementary Materials.

## 3. Results

Corpus characteristics are given in Table 1. The 13,073 audited meals came from 513 registrants over 179 days; 48.3% carried an attached photograph. Median energy per meal was 420 kcal (IQR 300–570).

### 3.1. The Engine Passes Every Conventional Quality Control

Reported energy and energy reconstructed from Atwater factors correlated at r = 0.9951 (95% CI 0.9950–0.9953; *n* = 13,029 meals with energy > 0). The median relative error was 0.0000 (IQR −0.0185 to +0.0233), and 96.2% of meals fell within ±10%. Internal plausibility constraints were essentially never violated: sugar exceeded carbohydrate in 1 of 13,073 records, fibre exceeded carbohydrate in 4, and no meal fell outside 0–4000 kcal (Table 2, tests T5 and T6).

Any conventional nutrition data-quality audit would pass this engine. That is the premise of this study, not an aside.

### 3.2. Terminal-Digit Distribution (T1)

**Figure 1:**
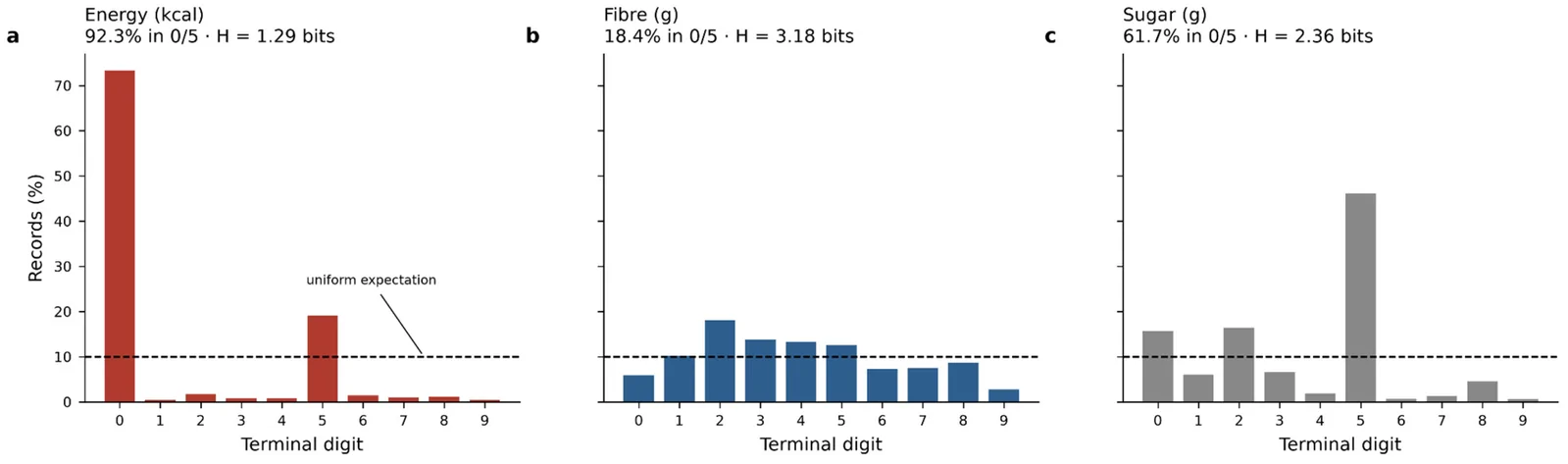
Terminal-digit distribution of engine-returned values (exact zeros excluded). The dashed line marks the uniform expectation of 10% per digit. (**a**) Energy (kcal): values are strongly concentrated on terminal digits 0 and 5 (92.3% in {0,5}; entropy H = 1.29 bits). (**b**) Fibre (g), an internal negative control: the distribution is near-uniform and compatible with a composition-table lookup (18.4% in {0,5}; H = 3.18 bits). (**c**) Sugar (g), exact zeros excluded (80.0% of sugar records are exactly zero): values concentrate on {0,5} (61.7%; H = 2.36 bits); including the exact zeros inflates the {0,5} share from 61.72% to 92.34%.

Energy is the extreme case: 92.29% of non-zero values end in 0 or 5, against the 20% expected under uniformity, with an entropy of 1.2900 bits out of a possible 3.3219 (38.8% of the available information) and only 762 distinct values across 13,073 meals (χ^2^ = 61,730, 9 df). All χ^2^ statistics fall below the smallest representable double-precision *p*-value and are reported as *p* < 0.001; an exact *p* adds nothing once a 9-df statistic exceeds 60,000 (Figure 1a; Supplementary Table S1).

Zero inflation is a trap, and sugar falls into it. Sugar shows an apparent 92.34% in {0,5}, but 80.0% of sugar records are exactly zero and a zero contributes trivially to digit 0. Excluding zeros, the indicator falls to 61.72%, and entropy rises from 1.0018 to 2.3584 bits (Figure 1c). The energy figure survives the same correction unchanged (92.31% → 92.29%), because only 0.34% of energy values are exactly zero.

Fibre is the expected exception and serves as an internal negative control. With zeros excluded, fibre falls to 18.43% in {0,5}—below the 20% uniform expectation—and carries the highest entropy of the six nutrients (3.1838 bits; Figure 1b). This is the behaviour of a composition-table lookup. Had the energy pattern been an artefact of the storage layer or the export pipeline, fibre would show it too.

### 3.3. Grid Adherence and Resolution (T2, T3)

First, 92.30% of energy values are exact multiples of 5, 73.34% of 10, and 29.61% of 25. Effective resolution is 762 distinct values across 13,073 records (5.83%). By contrast, fibre has only 138 distinct values but spans 0–36 g, so its relative granularity is far higher than that of energy (Supplementary Table S2).

### 3.4. The Engine Does Not Reproduce Itself (T4)

What follows quantifies the instability of the deployed pipeline under repeated occurrence of the same input. It does not isolate sampling stochasticity: because the model version, sampling temperature, and provider-side prompts were outside our control across the 179-day window, the within-string variance confounds estimator noise with model-version drift, and the design cannot separate them (Section 4.3). Both mechanisms make the instrument unusable without measurement, which is the claim this section supports; neither is established as the cause.

**Figure 2:**
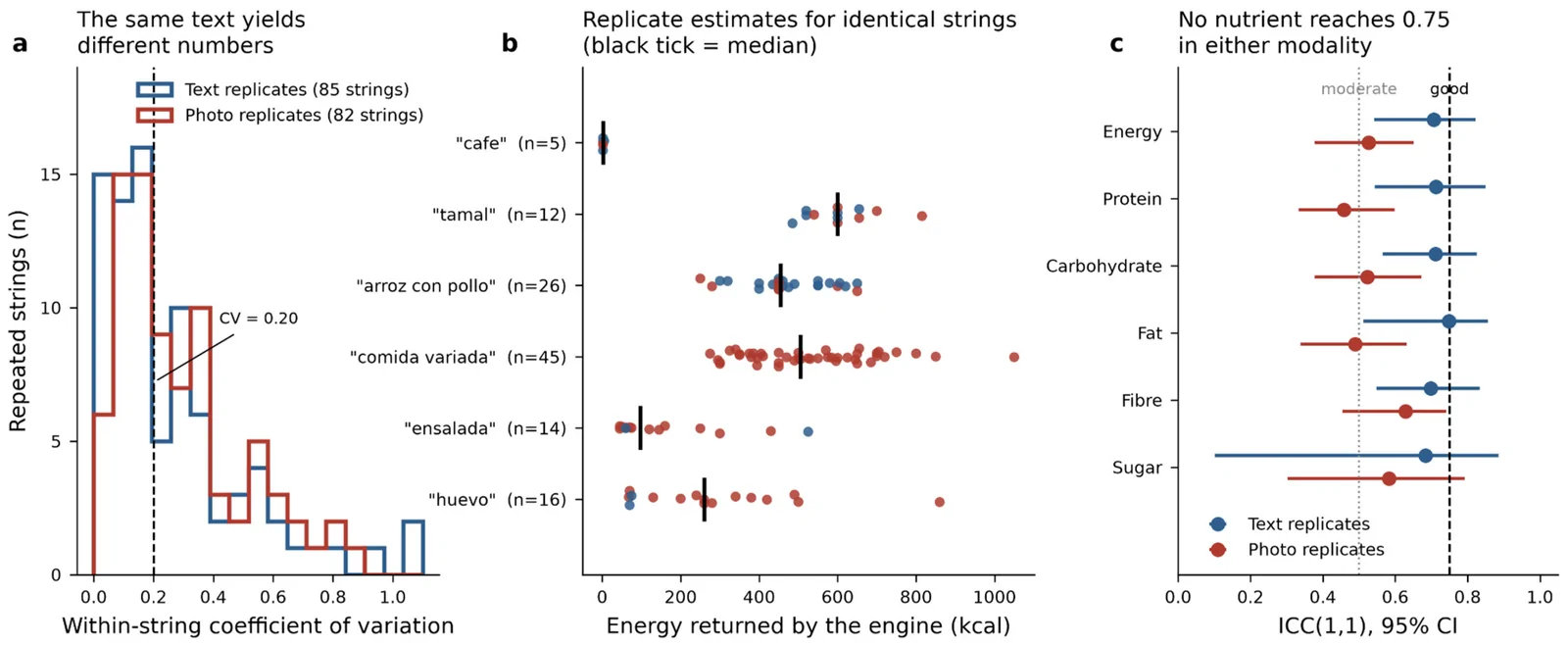
Test–retest reliability on recurring identical free-text strings (191 strings, 944 meals). (**a**) Within-string coefficient of variation by input modality. (**b**) Replicate estimates for six literal corpus strings; black tick marks the median; point colour indicates modality. (**c**) ICC(1,1) with 95% bootstrap confidence intervals by nutrient and modality stratum. Strata are defined at the level of the replicate: a string enters the text stratum if it has three or more text-only replicates and the photograph stratum if it has three or more photograph-accompanied replicates, so the two are not disjointed (Supplementary Table S11). No nutrient reaches the 0.75 threshold in either stratum. The between-stratum difference is not robust to specification (Supplementary Tables S7 and S8) and is reported as design control, not an effect.

Across 191 normalised strings repeated three or more times (944 meals), the median within-string coefficient of variation for energy was 0.2254; 54.45% of strings exceeded a CV of 0.20 and only 2.09% received an identical value every time (Figure 2a).

Stratified by input modality (Figure 2c; Table 3):

No nutrient reached the 0.75 threshold separating moderate from good reliability in either stratum. In the text-only stratum, the point estimates ranged from 0.6836 (sugar) to 0.7486 (fat); in the photograph stratum, it ranged from 0.4579 (protein) to 0.6289 (fibre). Upper confidence limits crossed 0.75 for several nutrients in the text-only stratum, so we do not claim the text-only estimate is established below the threshold—only that its point estimate is, and that the photograph stratum lies below it with the entire interval.

#### 3.4.1. The Modality Difference Is Not Robust

The apparent gap between strata for energy was ΔICC = 0.1803 (95% bootstrap CI −0.0164 to 0.3658; P(text > photo) = 0.964). We subjected it to three checks, and it did not survive them (Supplementary Tables S7 and S8):

- Repetition threshold. Lowering the inclusion threshold from three to two repetitions more than doubles the substudy (556 strings, 1674 meals) and attenuates the contrast to ΔICC = 0.0857 (95% CI −0.0746 to 0.2273), with the photograph stratum rising to 0.6122.
- Paired within-string comparison. The cleanest available test compares the two modalities inside the same string, which holds the food description exactly constant. Thirty strings had at least two replicates of each type. Median within-string CV was 0.1831 for text replicates and 0.2899 for photograph replicates, but the paired difference was null (Wilcoxon *p* = 0.7922; photographs more variable in 17 of 30 strings).
- Placeholder text. Excluding generic placeholder strings leaves the direction unchanged (0.5593 photo versus 0.7061 text) with identical median description length in both strata (7 words), so the gap is not an artefact of less informative text.

The direction is consistent across specifications, but the magnitude is not, and the paired test—the one with the strongest internal control—is null. We therefore do not claim a modality effect. What the data do establish is narrower and still consequential: because 86 of 191 repeated strings mixed submission types, a reliability coefficient computed without checking replicate comparability is uninterpretable. Modality is a design control that any such audit must apply, not a finding this study can report. A properly powered test is specified in Section 4.3.

Literal examples convey the magnitude better than a coefficient. “Huevo” (egg, *n* = 16) received between 68 and 860 kcal; “ensalada” (salad, *n* = 14) received between 45 and 525 kcal (Figure 2b; Supplementary Table S6).

#### 3.4.2. Reliability Depends Strongly on Description Specificity

Within-string variability decreased monotonically with description length (Spearman ρ between CV and word count = −0.5931, *p* = 1.57 × 10^−19^; Table 4):

This has a consequence favourable to the engine that must be stated. Repeated strings are over-represented at the non-specific end—“comida”, “ensalada”, “huevo” repeat because they are generic—whereas the full corpus is dominated by long descriptions (67.62% have six or more words). We do not report a single corpus-standardised coefficient. Standardising an ICC across strata cannot be done by averaging the stratum coefficients, because an ICC is a ratio of variance components rather than a quantity that combines linearly; and reconstructing it from the components requires assuming that each stratum’s between-string variance transfers unchanged to the corpus mixture, which these data do not establish. What the observed gradient supports is the direction, not a number: the pooled coefficient understates the reliability a corpus of longer descriptions would show and overstates the reliability of the short generic strings that recur most. The qualitative conclusion is unchanged either way: a user who types “huevo” receives an essentially arbitrary number within a 12-fold range.

A further sensitivity analysis (Supplementary Table S3) shows that strings submitted by a single registrant reach ICC 0.8535, whereas strings shared across registrants reach 0.5632. Single-registrant strings are longer and more descriptive; this is the specificity gradient seen from another angle, not a registrant effect. Raising the repetition threshold to five or ten occurrences leaves the estimate essentially unchanged (0.5953 and 0.6424).

#### 3.4.3. How Far the Replicates Fall Short of True Resubmission

Two properties of a retrospective design separate our replicate sets from the resubmission experiment the reliability coefficient would ideally rest on, and both are measurable rather than hypothetical.

Raw text. Replicate membership is defined on normalised text, so a set may contain records whose original strings differed in capitalisation or diacritics. Measured directly: 167 of the 191 strings (87.4%) contained a single raw form, covering 745 of the 944 replicates (78.9%). The remaining 24 sets differ only in case and accents—“Jugo de Maracuyá”, “Jugo de maracuyá”, “jugo de maracuyá”—with at most three raw forms in any one set. Restricting the analysis to the 167 byte-identical sets gives an energy ICC of 0.5985, against 0.5905 on all 191. The definition of identity moves the coefficient by less than a hundredth.

Photographs. This gap cannot be closed with our data. A photograph-accompanied record shares only its text with its replicates; the image differed on every occasion, and nothing in the operational record allows us to verify otherwise. Further, 506 replicates in 134 strings carry a photograph. For those, the engine received different multimodal inputs, so part of the within-string variance is the variation between meals and photographs rather than instability of the estimator. Reliability estimated on that stratum is therefore not test–retest reliability in the conventional sense, and we do not interpret it as such anywhere in this manuscript.

What survives both restrictions. Applying the strictest available definition of “the same input”—byte-identical raw text and no photograph in any replicate—leaves 74 strings and 298 replicates and gives an energy ICC of 0.6944. That estimate is free of both objections and still falls below the 0.75 threshold fixed in advance. The conclusion of this section does not depend on the permissive definition: it holds under the strictest one the corpus permits.

Which estimate is most representative of real-world use. Stating this explicitly matters, and we regard the text-only stratum with one record per registrant, ICC 0.6313, as the estimate most representative of typical real-world use of this pipeline. Our reasoning: it is the only figure that is simultaneously free of the multimodal-identity objection (Section 3.4.3), free of inflation by repeated logging from a single person (Section 3.4.4), and computed on inputs the engine genuinely received as the same text. It is also the most pessimistic of the defensible estimates, which is the direction an acceptance decision should err in. The pooled 0.5905 is less representative because it mixes input conditions. We report the full set because the spread is itself the finding—a single coefficient would misrepresent an instrument whose reliability depends this strongly on what it is asked.

#### 3.4.4. Agreement Is Not an Artefact of Repeated Logging by the Same Registrant

Multiple records per registrant raise the possibility that a coefficient is inflated by one person logging the same phrase in the same way, rather than by the engine behaving consistently. Retaining the first record per registrant per string removes that path entirely and lowers the estimate in all three strata (Table 5):

The direction is the one the objection predicts, and it works against the engine rather than for it: the unadjusted figures are the optimistic ones. No stratum reaches 0.75 after the adjustment, and the text-only estimate falls from 0.71 to 0.63. The same pattern appears from the other side in Supplementary Table S3, where strings logged by a single registrant reach 0.8535, while strings shared across registrants reach 0.5632—a gradient the manuscript attributes to description specificity (single-registrant strings are longer), and which this analysis shows is not merely a registrant effect, since removing within-registrant repetition lowers the coefficient even within strata.

### 3.5. Variance Decomposition

Estimator noise (MSW) was 15,601 kcal^2^, i.e., SD = 124.9 kcal, or 29.7% of the corpus median (420 kcal). True between-string variance was 22,500 kcal^2^. Within the repeated-string subset, 41.1% of the variance is estimator noise (MSW 15,601.1 kcal^2^ over the total variance of the repeated subset, 37,922 kcal^2^); expressed against the total variance of the full corpus (51,743 kcal^2^; SD 227.5 kcal), estimator noise accounts for 30.2%.

Noise is far from homogeneous: MSW was 24,634 kcal^2^ for one-to-two-word strings (SD 157 kcal), 10,782 kcal^2^ for three-to-five-word strings, and 1342 kcal^2^ for strings of six or more words (SD 37 kcal).

### 3.6. Quantified Attenuation

In the subcohort of 62 women with laboratory fasting glucose and at least five logged meals (median 19.5 meals, IQR 7.3–38.0), the harmonic mean was k^−^ = 12.55 meals per participant. With σ^2^_e_ = 15,601 kcal^2^, noise in the personal mean is 1243 kcal^2^ (SD 35.3 kcal) against an observed between-participant variance of 5922 kcal^2^ (SD 77.0 kcal), giving

λ_person = 0.79, and an attenuation factor for correlations of √λ = 0.889.

This calculation and everything that follows from it are a model-dependent sensitivity analysis rather than an empirical estimate of the underlying epidemiological relationship, and we present them as such. Applying a single within-string variance to participant means assumes homogeneous and mutually independent measurement errors across a participant’s meals. Neither assumption is demonstrated here, and one of them is contradicted by our own data: Section 3.4.2 shows the error variance depends strongly on description length, and Section 2.5 shows it differs by input modality, so a participant who logs mostly short descriptions carries more noise than λ implies and one who writes long ones carries less. Errors within a participant may also be correlated because the same person tends to describe food the same way, which would make the correction optimistic. The figure below should therefore be read as what the attenuation would be if the error structure were homogeneous, a bound useful for design, not as a corrected estimate of a diet–glycaemia association. We report a per-stratum range alongside it (Table 6) so the reader can see how much the assumption is doing.

This is the figure that governs the epidemiological analysis, and it is considerably higher than the meal-level ICC because averaging over meals reduces noise by a known factor.

**Figure 3:**
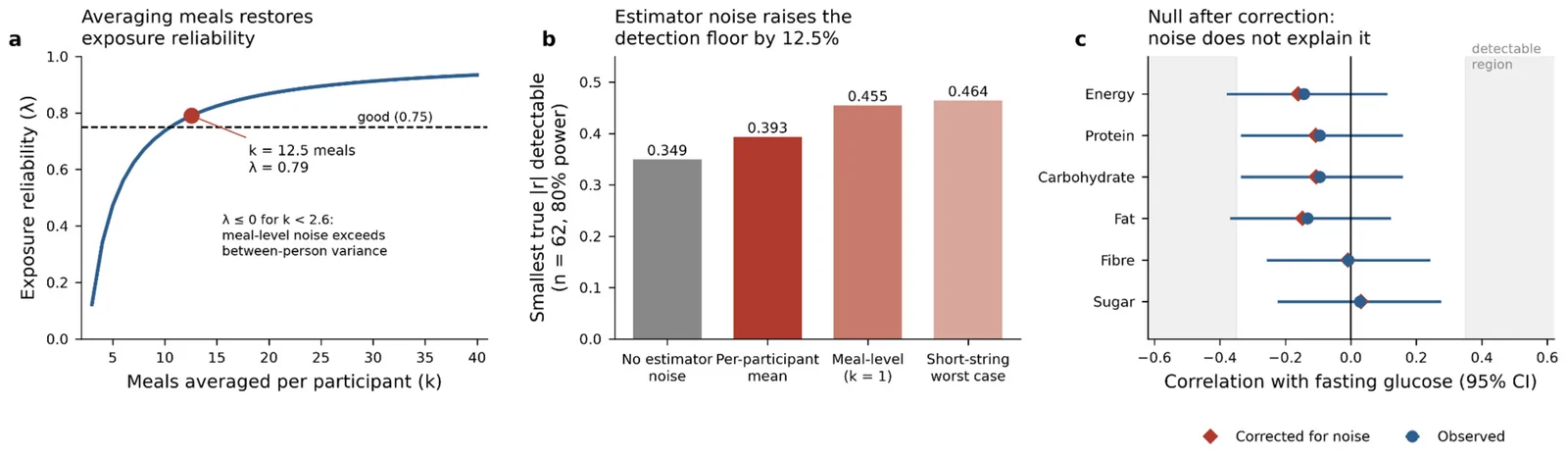
Quantified attenuation. (**a**) Exposure reliability as a function of the number of meals averaged per participant, with the observed operating point marked (k = 12.55, λ = 0.79). (**b**) Smallest detectable true correlation under four reliability scenarios (*n* = 62, 80% power). (**c**) Observed and noise-corrected diet–glycaemia correlations against the detection floor. Averaging restores reliability, but the associations remain null after correction: estimator noise does not account for the null findings.

With *n* = 62, the smallest correlation detectable at 80% power and α = 0.05 two-sided is |r| = 0.3494. Discounting engine noise, the smallest detectable true correlation rises to 0.3931 (+12.5%), and the effective sample size falls from 62 to 49.0 participants—engine noise costs the equivalent of 13 participants (Figure 3a,b; Table 7).

Observed diet–glycaemia associations were null across all six exposures (Figure 3c; Supplementary Table S5):

Correcting for instrument unreliability moves the largest association from |r| = 0.1430 to 0.161, which is still far below the 0.3494 detection floor. Estimator noise therefore does not account for the null. It raises the detection threshold by 12.5%; the available sample size is the dominant constraint, since detecting a true r of 0.30 would require 109 participants and only 62 were available. The instrument is irreproducible and the study is underpowered; neither problem rescues the other, and quantification tells us exactly what each costs.

### 3.7. Specificity of the Terminal-Digit Test

**Figure 4:**
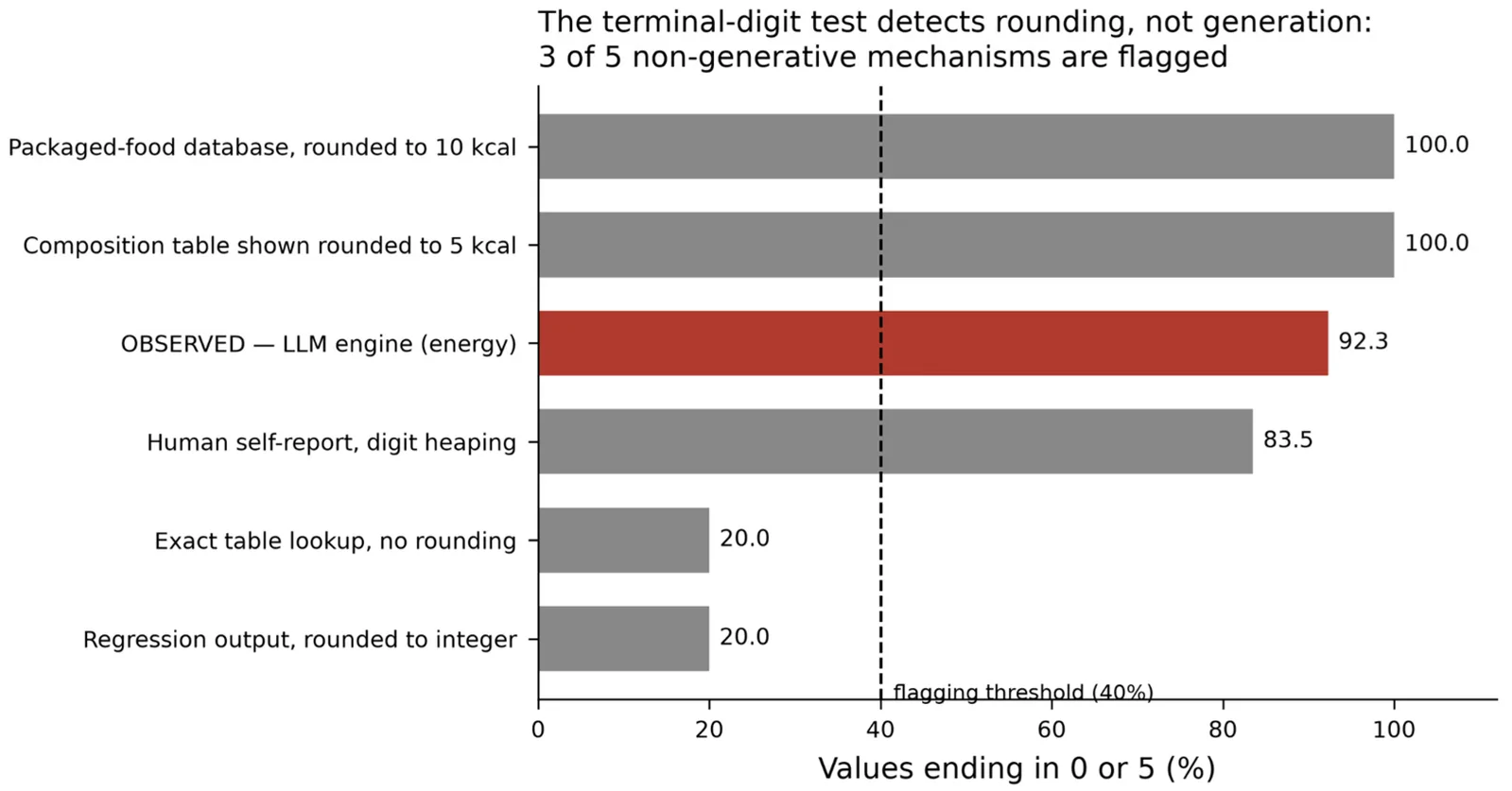
Specificity of the terminal-digit test. Three of five simulated non-generative data-producing mechanisms are flagged at the pre-specified threshold. The test identifies coarse rounding, not the mechanism that produced the numbers; the reliability test (Figure 2) carries the causal weight of the argument.

Five simulated non-generative mechanisms were passed through the same threshold (Figure 4; Supplementary Table S4).

Three of five were flagged, including two entirely legitimate scenarios: a composition table displayed with rounding (100% in {0,5}) and human self-report with digit heaping (83.5%). An exact table lookup without rounding (20.00%, H = 3.3218) and a rounded regression output (19.98%, H = 3.3213) were not flagged.

The test therefore detects rounding, not generation. This is why T1 is not the decisive test of this paper: the causal weight rests on T4, which no deterministic table lookup can fail.

### 3.8. Silent Failure of the Vision Stage Degrades the Text the Second Stage Receives

The two-stage architecture (Section 2.2) implies that for photograph-accompanied entries the description reaching the nutrient-estimating stage is written by the vision model rather than by the participant. A post-hoc comparison of the two strata confirms this and identifies a failure mode invisible to every conventional quality control (Supplementary Table S12).

The most frequent descriptions differ in kind, not merely in wording. Among text-only entries they are ordinary Spanish food phrases (arroz con pollo, *n* = 18; pan y cafe sin azucar, *n* = 9). Among photograph-accompanied entries, the three most frequent are comida variada (*n* = 45), comida (*n* = 16), and comida analizada (*n* = 16), the last of which is not a food but an artefact of the vision stage, emitted when it fails to identify the photograph’s contents. Counting a pre-specified list of such artefact strings, 1.44% of photograph-accompanied records (91/6320) carry a vision-stage failure label, against 0.01% of text-only records (1/6753); odds ratio 98.6, and 98.9% of all artefact strings in the corpus accompany a photograph.

These strings behave as near-pure noise. The five artefact strings that entered the test–retest substudy contribute 90 of its 944 meals (9.5%), with an ICC for energy of 0.0911 within the photograph stratum (89 meals) and 0.1480 across both strata (90 meals) against 0.5905 for the substudy as a whole. Comida variada received 45 values spanning 275 to 1050 kcal; desconocido spanned 0 to 550 kcal (within-string CV 0.97).

Excluding artefact strings raises reliability without approaching the threshold: the photograph stratum moves from 0.5258 to 0.5720, and the pooled estimate moves from 0.5905 to 0.6088. The primary conclusion is therefore unchanged, and the mechanism is now partly identified: a component of what a single-stage reading would attribute to estimator noise is silent failure of the upstream stage, which degrades the second stage’s input instead of returning an error. The pipeline does not report that the photograph could not be read; it emits a generic phrase, and the second stage obediently assigns it a number. For any team auditing a multimodal cascade, this is the more general lesson: an identifiable failure mode is not numerical error but silent degradation of the interface between stages, and it is detectable only by inspecting the intermediate representation.

## 4. Discussion

### 4.1. Principal Findings

These words are used differently across the fields this work touches, so we fix them operationally before interpreting anything. Reproducibility here means agreement between values the pipeline returns for the same input on separate occasions, which is what T4 measures. Reliability is the ratio of true to total variance in an intended analytical unit, estimated as ICC at the record level and as λ at the participant level. Validity—whether the numbers are correct—is not assessed anywhere in this study, and cannot be: without a reference standard, accuracy and criterion validity are outside its reach. A pipeline can be perfectly reproducible and entirely wrong, or accurate on average and useless per record. What we show is that the conventional controls speak to neither reproducibility nor validity, and that the cheap test that does speak to reproducibility is failed.

A deployed LLM nutrition engine passed every quality control a conventional audit would apply—Atwater coherence at r = 0.9951, a median relative error of exactly zero, one plausibility violation in 13,073 records—and failed the one control that is almost never applied: it does not return the same number when shown the same input. On text-only recurring strings, the intraclass correlation for energy was 0.7061, below the 0.75 threshold for good reliability; when a photograph accompanied the text, it was 0.5258, with the entire confidence interval below that threshold.

The practical statement is blunt. A participant who types “huevo” receives a number drawn from a range spanning 68 to 860 kcal. No amount of downstream statistical care repairs an exposure measured that way at the level of a single meal.

A further finding locates part of the mechanism. Because the pipeline is a cascade, the description reaching the nutrient-estimating stage is written by the vision stage whenever a photograph is attached, and 1.44% of those descriptions are not food phrases at all but failure artefacts such as comida analizada, against 0.01% of participant-written entries (Section 3.8). Those strings are near-pure noise (ICC 0.0911), and excluding them raises the photograph-stratum estimate from 0.5258 to 0.5720 without approaching the threshold. The upstream stage does not signal that it could not read the photograph; it emits a generic phrase, and the downstream stage assigns it a number. This reframes what a reliability audit of a cascade must inspect: the intermediate representation is where the failure becomes visible, and a pipeline audited only at its output will attribute to stochasticity what is in fact an unreported error path.

Three further findings temper and sharpen this. First, reliability improves steeply with description specificity: one-to-two-word strings reach only 0.5668, while strings of six or more words reach 0.9181, a gradient that is observed rather than modelled. Second, averaging meals within a participant substantially repairs the exposure: λ rises to 0.79 at 12.55 meals, so cohort analyses using per-participant means are far less compromised than the meal-level coefficient suggests. Third—and this is why the audit must be reported honestly rather than dramatically—engine noise does not explain the null diet–glycaemia associations in this cohort. It raises the detection floor by 12.5%; the sample size does the rest.

A fourth observation concerns method rather than result. Nearly half the records carried a photograph, and 86 of 191 repeated strings mixed submission types, so the pooled coefficient that a naive audit would report conflates two input conditions. Stratifying separated them, but the resulting difference did not survive a change of repetition threshold or a paired within-string test. We report modality as a control that must be applied, not as an effect we have demonstrated. This distinction matters for anyone replicating the battery: the check is mandatory, its result here is inconclusive, and the inconclusiveness is itself informative about how much data a modality comparison needs.

### 4.2. Comparison with Existing Literature

Published evaluations of LLM nutrition estimation are accuracy studies against a reference standard, and all issue a single invocation per item. Where they report an ICC, it quantifies agreement between the model and human assessors or a database, not agreement of the engine with itself. A targeted PubMed query crossing nutrient estimation with the intraclass correlation coefficient returns seven records, none of which measures intra-engine reproducibility on repeated inputs.

Instability for recurring identical input is not hypothetical. Repeated queries of the same medical question return materially different answers across sessions [38]; the same non-determinism has been quantified outside medicine, where a fixed prompt issued repeatedly to a commercial model yields outputs that differ in substance and not only in wording [39]; and the mechanism by which a generative model produces a confident but unfounded value has itself become an object of measurement [40]. None of this work concerns nutrient estimation, and none of it produces a reliability coefficient on a continuous quantity, but together it establishes that variability on recurring identical input is a property of the technology, not an artefact of one deployment.

The resubmission design is established elsewhere in medicine, but always with categorical or ordinal output, and always with text input. To our knowledge, this is the first reliability audit of an LLM-assisted nutrition engine on naturally recurring identical text in its operational record, and the first application of terminal-digit analysis—previously confined to human observers using physical instruments—as a quality control on the numbers a generative estimator returns. Terminal-digit and related distributional screens are established tools for auditing survey data collected by humans, where investment in measurement quality demonstrably shifts the digit distribution [41,42]; applying them to machine-generated values extends a survey-methodology instrument to a new source of numbers. The broader concern they serve—that analyses can look sound while resting on a data-generating process nobody inspected—is the same one that has driven reporting reform in machine-learning-based science [43].

Placing the coefficient in context requires the dietary-assessment literature because nutritional epidemiology has spent decades characterising a different error source in the same measurement. Self-reported instruments carry an error that is neither small nor random: it is correlated with body size, with the reporting instrument itself and with the intake being reported [44,45]. Energy intake obtained by 24 h recall or food-frequency questionnaire underestimates expenditure measured by doubly labelled water by margins large enough to bias associations [46,47,48], and the reference method itself carries an appreciable coefficient of variation [49]. Online self-administered recalls—the closest methodological relatives of a conversational logging agent—have been evaluated for both validity and repeatability and are reported with test–retest coefficients alongside their accuracy [50]. Our finding sits beside this body of work rather than inside it: the informant error it describes is additional to the estimator error we measure, not a substitute for it, and an engine that is unreliable when the same input recurs adds a second variance component to a measurement that was already noisy.

The measurement-error framework we use to translate unreliability into power is long established in nutritional epidemiology, but it models error in the human informant. The error documented here arises in the algorithmic estimator, is independent of the informant, and—unlike human error—costs nothing to measure. That last property is the practical contribution: any team integrating a third-party nutrition engine can run this battery on its own production data in an afternoon.

We could locate no study stratifying the reliability of a nutrition engine by input modality: sixteen queries across PubMed and OpenAlex returned no such work, and the two most direct formulations of the contrast returned zero relevant hits. We state this as absence of retrieved evidence rather than demonstrated absence—grey literature, non-indexed proceedings and computer-science preprints were not covered—and we emphasise that our own data do not fill the gap. They show that the comparison is necessary and that this corpus cannot settle it.

### 4.3. Limitations

1. No reference standard. No 24 h recall, weighed record, or doubly labelled water was collected. This study cannot claim the engine’s numbers are wrong on average; it claims they are not reproducible, which is a distinct and necessary property. A biased but reproducible engine would be calibratable; an irreproducible one is not. Establishing accuracy would in any case require a reference the engine itself lacks: composition databases are incomplete, differ between countries and carry their own missing-value and coverage problems [51,52], and regional intake studies have documented how poorly the food supply of Latin American populations maps onto the sources such databases were built from [53].
2. The modality contrast is not established, and we do not claim it. The bootstrap interval for ΔICC crosses zero (−0.0164 to 0.3658), the contrast attenuates to 0.0857 when the repetition threshold is lowered, and a paired within-string comparison is null (*p* = 0.7922). The direction is consistent, but the effect is not demonstrated. Modality enters this manuscript as a mandatory design control on replicate comparability, not as a result.
3. The terminal-digit test detects rounding, not generation. Three of five simulated non-generative mechanisms are flagged at the same threshold, including two legitimate ones. T1 is a screen, not proof; the causal weight rests on T4.
4. Replicates did not arise from a re-test protocol. Repetitions of an identical string occurred naturally, separated in time and sometimes originating from different registrants. This is also why the photograph stratum cannot be interpreted as an evaluation of image-based estimation as such: purpose-built image-based dietary assessment is a distinct methodological literature with its own validation designs and error characteristics [54,55,56], and what we observe here is a general-purpose engine receiving an unstandardised photograph alongside free text, not a system designed for portion estimation from images. Neither the underlying model version, nor sampling temperature, nor possible provider-side prompt changes were controlled across the 179-day period. Part of the within-string variance may reflect version drift rather than sampling stochasticity—the behaviour of a commercial model on a fixed prompt has been shown to change materially between releases [57]—two mechanisms with different implications that this design cannot separate. The experiment that would resolve both this and the modality question is cheap, and we specify it explicitly: resubmit 200 texts in a single session against a fixed model version, each submitted twice with text alone and twice with an accompanying photograph, giving a paired design with roughly 10 times the effective sample of the 30 mixed strings available here.
5. The pooled ICC depends on the text mixture. Repeated strings over-represent short generic descriptions, which are the least reliable, so the pooled coefficient is specific to the mixture of strings that happened to recur and is not a corpus-wide reliability. We report it per stratum rather than standardising to the corpus because no standardisation is available that does not require an assumption about the transfer of between-string variance that these data cannot support.
6. Residual test contamination. The es_prueba flag is null for 158 registrants, so an unknown number of test records may remain. Two identifier keys were needed to detect flagged accounts because neither covers the corpus alone.
7. Statistical power of the bridge. With 62 women the analysis detects only |r| ≥ 0.3494 at 80% power, well above plausible diet–glycaemia associations. The null is reported as a null finding, and a null in this sample is uninformative about associations below that magnitude.
8. Limited generalisation. The corpus is rural Colombian Spanish, with Andean dishes (arepa, sancocho, changua) sparsely represented in the English-language composition databases the model was likely trained on. Irreproducibility may be greater here than in an English corpus of standard foods. The battery is portable; the magnitude of the finding is not.
9. The cascade cannot be pinned, and its two stages are confounded. The model families are known, but prompts, temperature, checkpoints, and any composition-database anchoring are provider-controlled and revised without notice, so the audited object is the deployed cascade at the checkpoints in force during the window rather than a fixed estimator. Because photograph-accompanied entries pass through both stages while text-only entries pass through the second alone, and because the first stage alters the distribution of the text the second receives (Section 3.8), the two stages are not separable in this retrospective design: the difference between strata cannot be read as the contribution of the vision stage. Separating them requires resubmission against pinned checkpoints with the intermediate text frozen, which the acceptance test specifies (Section 4.4). This limitation is representative of any team integrating third-party services and is precisely the scenario the battery is designed for.
10. A single deployment. One corpus, one provider pair, one period. Replication in other pipelines is necessary, and the battery is published to make it possible.

### 4.4. Implications

For teams deploying nutrition pipelines, the recommendation is concrete: resubmit a sample of identical inputs and compute an ICC before trusting the output, stratifying by input modality. The test costs the price of a few hundred API calls and is, in this evidence, the binding constraint on data quality—not accuracy against a reference standard, which is both more expensive and, in a deployed system, usually impossible.

Because the value of a reproducibility check lies in it being run by others, we state the procedure as a portable acceptance test rather than as a description of what we did. We refer to it as the repeated-input reliability audit (REPRA) so that it can be cited and requested by name. It requires no reference standard, no additional data collection, and no access to the model:

What REPRA is and is not, at this stage. It is a hypothesis-generating proposal rather than an established or validated standard, and the specific parameters carry no evidential backing from this study. The sample size of 100 inputs is a pragmatic floor, not a figure derived from a power calculation for reliability estimation; the threshold of 0.75 is the convention discussed in Section 2.6, not a value shown to separate fit-for-use pipelines from unusable ones; and the recommendation of three resubmissions reflects the minimum that makes a within-input variance estimable, not an optimum. Nothing in our single-deployment audit establishes that a pipeline passing REPRA is fit for use, only that one failing it returns numbers a downstream analysis cannot rely on. Validating the procedure itself—calibrating the sample size, testing whether the threshold predicts anything about downstream inference, and checking that the verdict is stable across deployments—requires the multi-engine benchmark we describe as future work, not this corpus. We publish the protocol because a procedure that is named and specified can be requested, criticized, and improved, which an unnamed practice cannot.

1. Sample at least 100 distinct inputs from production traffic, drawn to match the length and modality mixture of the real corpus rather than a convenience subset; reliability rose from 0.5668 to 0.9181 across length bands in our data, so a sample skewed toward long descriptions will overstate the engine.

2. Resubmit each input at least three times within a single session against a pinned model version, recording the version identifier. Separating replicates in time confounds sampling variability with version drift, as our own design does.

3. Hold the modality constant within an input and submit text-only and photograph-accompanied variants as separate arms. Mixing them within a replicate set makes the resulting coefficient uninterpretable; 86 of 191 repeated strings were mixed in this corpus before stratification.

For the photograph arm, this requires resubmitting the identical image file alongside the identical text, not a new photograph of the same dish: an input is multimodal, and holding only the text fixed leaves the image varying, which is precisely the limitation of our own retrospective estimate (Section 3.4.3). A prospective audit can meet this condition trivially by storing and replaying the original file; a retrospective one cannot recover it, which is why the photograph stratum reported here is not test–retest reliability in the conventional sense.

4. Compute ICC(1,1) with a bootstrap interval over inputs, not over rows, and report it per nutrient rather than for energy alone.

5. Decide against a threshold fixed in advance. We used 0.75; a programme whose analytic target is a per-participant mean may justify a lower meal-level threshold, but it should then report the harmonic mean of records per participant and the implied exposure reliability λ because that is what determines whether the instrument can support the intended inference.

6. Screen the output distribution for grid adherence and terminal-digit concentration. This is a cheap secondary check, and, as our specificity simulation shows, it flags legitimate rounding as readily as generation—it localises where to look and cannot on its own establish how the numbers were produced.

7. If the pipeline is a cascade, audit the intermediate representation and freeze it in one arm. Log the text (or other intermediate output) each stage passes downstream and report the prevalence of failure artefacts in it as an outcome in its own right. In this corpus, that prevalence differed 98.6-fold between strata and exposed an error path no output-level check detected. To separate the stages rather than merely observe their sum, add an arm in which the intermediate text produced once by the upstream stage is frozen and resubmitted k times to the downstream stage: the contrast against an arm that resubmits the original photograph isolates the upstream stage’s contribution. Without that arm—as in our own retrospective design—the stages are confounded and a difference between modality strata cannot be attributed to either.

Our retrospective design could execute steps 1, 4, 5, and 6, and step 7 only in its diagnostic half; we inspected the intermediate text but could not freeze and replay it. Steps 2 and 3 are the parts the design could not satisfy: the model version was not pinned, and for photograph-accompanied records, the image was not the same file. Those two omissions are the reason two of our findings remain unresolved, and they are why the protocol is offered as a prospective procedure rather than as something this study completed. Reporting the six items above alongside a deployed instrument would cost an afternoon and would make the reliability of that instrument comparable across studies, which is currently impossible.

For journals and reviewers, prospective test–retest reproducibility on deliberately resubmitted identical inputs should become an expected item in the evaluation of any LLM-based measurement instrument that returns continuous values. It is currently absent from the literature.

For cohort studies using such instruments, the attenuation arithmetic in Section 3.6 is directly reusable: report the harmonic mean of records per participant, the estimator’s within-record variance, and the resulting λ, so that readers can judge how much of a null is instrumental and how much is sample size.

## 5. Conclusions

Internal consistency does not validate an LLM-assisted nutrition pipeline. An engine can reconstruct its own energy values from its own macronutrients almost perfectly and still fail to return the same number when the same text recurs. What this retrospective design establishes is instability of the deployed pipeline under repeated occurrence of the same or near-identical input; it does not separate estimator noise from model-version drift, and for photograph-accompanied records, the replicates are not identical multimodal inputs at all because the image differed each time. Under the strictest definition the corpus permits—byte-identical raw text and no photograph—the energy coefficient is 0.6944, still below the pre-specified threshold, so the finding survives both restrictions. The prospective form of the test, resubmitting a chosen sample of identical inputs against a pinned model version with arms held constant, is the design that would attribute the variance; it is cheap, necessary, currently unreported, and specified step by step in Section 4.4. Where inputs may be multimodal, replicate comparability must be verified before any reliability coefficient is interpreted: in this corpus, 45% of the repeated strings mixed submission types, and stratifying them changed the headline number by more than a tenth of its value.

## Figures and Tables

**Table 1:** Corpus characteristics, distribution of engine-returned nutrient values, and composition of the test–retest reliability substudy. Registrant counts use the primary registration key of the patient registry (populated for 49.6% of records; shared as the pseudonymised registrant_id); the two alternative registry keys yield 638 and 255 distinct registrants, and no inference in this paper depends on the participant count.

Domain	Variable	Value
Corpus	Meal records, *n*	13,073
Corpus	Distinct registrants (identification key), *n*	513
Corpus	Distinct normalised free-text strings, *n*	11,955
Corpus	Date range	9 February 2026 to 6 August 2026
Corpus	Meals per registrant, median (IQR)	4 (1–12)
Corpus	Meals per registrant, range	1–229
Corpus	Records with an attached photograph, *n* (%)	6320 (48.3%)
Corpus	Records excluded as test accounts, *n*	868 (836 in window)
Corpus	Records excluded as re-ingestions, *n*	125
Estimated nutrients	Energy (kcal), median (IQR)	420.0 (300.0–570.0)
Estimated nutrients	Energy (kcal), mean (SD)	450.6 (227.5)
Estimated nutrients	Energy (kcal), min–max	0.0–2285.0
Estimated nutrients	Protein (g), median (IQR)	25.0 (15.0–35.0)
Estimated nutrients	Protein (g), mean (SD)	26.6 (15.3)
Estimated nutrients	Protein (g), min–max	0.0–280.0
Estimated nutrients	Carbohydrate (g), median (IQR)	45.0 (26.0–65.0)
Estimated nutrients	Carbohydrate (g), mean (SD)	48.4 (31.0)
Estimated nutrients	Carbohydrate (g), min–max	0.0–284.0
Estimated nutrients	Fat (g), median (IQR)	15.0 (10.0–21.0)
Estimated nutrients	Fat (g), mean (SD)	16.9 (11.7)
Estimated nutrients	Fat (g), min–max	0.0–140.0
Estimated nutrients	Fibre (g), median (IQR)	4.0 (2.0–7.0)
Estimated nutrients	Fibre (g), mean (SD)	5.2 (4.1)
Estimated nutrients	Fibre (g), min–max	0.0–36.0
Estimated nutrients	Sugar (g), median (IQR)	0.0 (0.0–0.0)
Estimated nutrients	Sugar (g), mean (SD)	2.0 (5.7)
Estimated nutrients	Sugar (g), min–max	0.0–108.0
Meal type	almuerzo	6125 (46.9%)
Meal type	desayuno	4023 (30.8%)
Meal type	cena	2924 (22.4%)
Meal type	snack	1 (0.0%)
Portion unit (top 6)	plato	9581 (73.3%)
Portion unit (top 6)	unidad	2389 (18.3%)
Portion unit (top 6)	porcion	427 (3.3%)
Portion unit (top 6)	desayuno	108 (0.8%)
Portion unit (top 6)	portion	105 (0.8%)
Portion unit (top 6)	taza	105 (0.8%)
Reliability substudy	Repeated normalised strings (≥3), *n*	191
Reliability substudy	Meals in repeated strings, *n*	944
Reliability substudy	Repeats per string, median (range)	4 (3–45)
Reliability substudy	Text-only repeated strings, *n*	85
Reliability substudy	Photo-accompanied repeated strings, *n*	82
Attenuation subcohort	Women with fasting glycaemia and ≥5 meals, *n*	62
Attenuation subcohort	Meals per participant, median (IQR)	20 (7–38)
Attenuation subcohort	Fasting glucose mg/dL, median (IQR)	88 (83–99)
Attenuation subcohort	Harmonic mean meals per participant	12.55
Beyond these three rules, the audit deliberately does not clean the corpus: removing outliers before an integrity test destroys the signal the test exists to detect. One pre-specified conditional analysis is reported with and without exact zeros, because zero inflation distorts the terminal digit (Section 3.2).		

**Table 2:** The six-test forensic battery: what each test measures, its statistic, the threshold fixed in advance of examining this corpus, the observed result, and the resulting verdict. Thresholds for T1–T3 come from the digit-preference literature and those for T4 from conventional reliability cut-points.

Test	Name	What it Measures	Statistic	Pre-Specified Threshold	Observed Result	Verdict
T1	Terminal digit	Output granularity	% digits in {0,5}; entropy H; χ^2^ (9 df)	Flag if %{0,5} > 40 or H < 2.50	92.29% · H = 1.2900 of 3.3219 bits · χ^2^ = 61,730	FLAGGED
T2	Grid adherence	Systematic rounding	% exact multiples of 5, 10, 25	Flag if multiples of 5 > 50%	5: 92.30% · 10: 73.34% · 25: 29.61%	FLAGGED
T3	Distinct values	Effective resolution	*n* distinct/*n* records	Flag if < 10%	762/13,073 = 5.83%	FLAGGED
T4a	Test–retest, text-only recurring inputs	Reproducibility	ICC(1,1), 95% bootstrap CI	Flag if ICC < 0.75	ICC 0.7061 (0.5419–0.8217)	FLAGGED (CI crosses the threshold)
T4b	Test–retest, photograph-accompanied recurring inputs	Reproducibility	ICC(1,1), 95% bootstrap CI	Flag if ICC < 0.75	ICC 0.5258 (0.3778–0.6513)	FLAGGED
T5	Atwater coherence	Internal energy coherence	Pearson r vs 4·carb + 4·prot + 9·fat	Flag if r < 0.95	r = 0.9951 (0.9950–0.9953); median relative error 0.0000	PASS
T6	Plausibility constraints	Internal consistency	Sugar ≤ carbohydrate; fibre ≤ carbohydrate; 0 ≤ kcal ≤ 4000	Flag if any constraint is violated in ≥1% of records	1 sugar > carbohydrate; 4 fibre > carbohydrate; 0 out of range (0.04% combined)	PASS
Test	Name	What it measures	Statistic	Pre-specified threshold	Observed result	Verdict

T1—Terminal digit. For each value, the last digit of the rounded number. We report the share falling in {0,5}, Shannon entropy H, and a χ^2^ statistic against uniformity (9 df). Flag if {0,5} > 40% or H < 2.50 bits. Reported with and without exact zeros. T2—Grid adherence. Share of values that are exact multiples of 5, 10, and 25. Flag if multiples of 5 exceed 50%. T3—Distinct values. Count of distinct values divided by record count. Flag if below 10%. T4—Test–retest on recurring inputs. Free text was normalised (lower-cased, accents and punctuation stripped, whitespace collapsed); strings occurring three or more times entered the substudy. Reliability was estimated as ICC(1,1), one-way random effects, unbalanced design [32,33]. Flag if ICC < 0.75. T5—Atwater coherence. Pearson correlation between reported energy and 4·carbohydrate + 4·protein + 9·fat—the general Atwater factors, whose accuracy limits are themselves documented [34]—plus the median relative error. Pass if r > 0.95 and |median relative error| < 0.10. T6—Plausibility constraints. Counts of sugar > carbohydrate, fibre > carbohydrate, and energy outside 0–4000 kcal. Pass if each violation affects under 1% of records.

**Table 3:** Test–retest reliability of energy by input-modality stratum. Strata are defined at the level of the replicate, so a string contributing replicates of both kinds enters both strata and the two are not disjointed (Table S11). ICC(1,1), one-way random effects, unbalanced design; 95% confidence intervals from 1000 bootstrap resamples over strings.

Stratum	Strings	Meals	ICC(1,1) Energy	95% CI
Text only	85	352	0.7061	0.5419–0.8217
Photograph attached	82	427	0.5258	0.3778–0.6513
Pooled (not interpretable as a single reliability coefficient)	191	944	0.5905	0.4962–0.6866

**Table 4:** Reliability of energy by description-length band, with each band’s share of the full corpus. Bands are reported separately rather than standardised to the corpus mixture: an ICC is a ratio of variance components and cannot be combined across strata by averaging the coefficients, and reconstructing one from the components would require assuming that each band’s between-string variance transfers unchanged to the full corpus, which these data do not establish.

Stratum	Strings	Meals	Median CV	ICC(1,1)	95% CI	% of Full Corpus
1–2 words	57	366	0.3982	0.5668	0.3906–0.6855	4.57%
3–5 words	110	485	0.2044	0.6243	0.4867–0.7305	27.81%
≥6 words	24	93	0.1015	0.9181	0.8248–0.9463	67.62%

**Table 5:** Test–retest reliability of energy before and after retaining a single record per registrant per string. The string and meal counts are those of the adjusted analysis; the unadjusted counts are 85 strings and 352 meals for text only, 82 and 427 for photograph-accompanied records, and 191 and 944 pooled. Δ is the change in ICC.

Stratum	ICC, All Replicates	ICC, One Record per Registrant	Strings, Adjusted	Meals, Adjusted	Δ
Text only	0.7061	0.6313	50	196	−0.0748
Photograph attached	0.5258	0.5004	60	311	−0.0255
Pooled	0.5905	0.5479	129	634	−0.0426

**Table 6:** Exposure reliability λ recomputed under the description-length strata of Section 3.4.2, holding the harmonic mean (k^−^ = 12.55) and the between-participant variance (5922 kcal^2^) fixed. The pooled λ = 0.79 sits inside a range that runs from 0.67 to 0.98 depending on which stratum a participant’s logging habits resemble, which is the quantitative form of the homogeneity assumption the pooled figure requires.

Length Stratum	Strings	Replicates	ICC(1,1) Energy	MSW (kcal^2^)	λ, This Stratum	Attenuation √λ
1–2 words	57	366	0.5668	24,633.8	0.6685	0.8176
3–5 words	110	485	0.6243	10,781.8	0.8549	0.9246
≥6 words	24	93	0.9181	1342.2	0.9819	0.9909
Pooled (reported above)	191	944	0.5905	15,601.1	0.7901	0.8889

λ, this stratum is the exposure reliability that would apply if every meal a participant logged resembled that stratum; k^−^ and the between-participant variance are held at their pooled values throughout.

**Table 7:** Correlations between per-participant mean nutrient intake and laboratory-measured fasting glucose in the attenuation subcohort (*n* = 62), observed and corrected for estimator unreliability (λ = 0.79). No association reaches the detection floor of |r| = 0.3494 (80% power, α = 0.05).

Exposure (per-Participant Mean)	r	95% CI	*p*	r Corrected (λ = 0.79)
Energy	−0.1430	−0.3792 to 0.1108	0.2677	−0.1608
Fat	−0.1318	−0.3694 to 0.1220	0.3072	−0.1483
Protein	−0.0951	−0.3368 to 0.1585	0.4623	−0.1070
Carbohydrate	−0.0946	−0.3364 to 0.1589	0.4646	−0.1064
Fibre	−0.0080	−0.2573 to 0.2422	0.9506	−0.0090
Sugar	0.0278	−0.2236 to 0.2756	0.8304	0.0312

## Data Availability

The shared corpus carries no direct identifier. Participants are represented by a registrant_id produced by keyed hashing of the internal registration key with a random salt that was not retained, so the pseudonym cannot be reversed or linked back to the registry by us or by anyone else; the original registration and document-number fields are absent from the shared file. The pseudonym is stable within the corpus, which is what the reported counts and the per-participant aggregation require, and it carries no information about the person. Meal descriptions are the participants’ own free text in Spanish and are shared verbatim because they are the object of study; they were screened and contain no names, contact details, or other personal information. The frozen audit corpus, all result tables, the number-to-source map, and the figure-generating code are provided as Supplementary Materials, together with the analysis script implementing the six-test battery so that the REPRA acceptance test in Section 4.4 can be run on other corpora without reimplementation. Readers should note which results the shared corpus does and does not regenerate. Every finding in Section 3.1, Section 3.2, Section 3.3, Section 3.4, Section 3.5, Section 3.7 and Section 3.8—the six-test battery, the ICC estimates and intervals, the variance decomposition, the specificity gradient and the artefact-string analysis—is fully reproducible from the frozen corpus alone. The attenuation bridge in Section 3.6 is not: it requires fasting glycaemia, sex, age, and diagnosis from the clinical registry, which cannot be shared owing to participant confidentiality, so the shared corpus contains meal-level fields only. To keep Section 3.6 auditable, we additionally provide a minimal de-identified derivative of the 62-participant subcohort (per-participant nutrient means, fasting glycaemia and the number of contributing records, with no identifier), which is sufficient to recompute the six reported correlations and the attenuation arithmetic. Individual-level registry data beyond that derivative cannot be shared.

## Supplementary Materials

The following supporting information can be downloaded at https://www.mdpi.com/article/10.3390/nu18193233/s1. Table S1: Terminal-digit distribution by nutrient, with and without exact zeros; Table S2: Grid adherence—share of values that are exact multiples of 5, 10, and 25; Table S3: ICC sensitivity to the repetition threshold and to the exclusion of artefact strings; Table S4: Specificity analysis—five simulated non-generative data-producing mechanisms; Table S5: Attenuation bridge—per-participant correlations with fasting glycaemia and noise-corrected estimates; Table S6: Attenuation scenarios, detection thresholds and required sample size; Table S7: Robustness of the modality contrast across three specifications; Table S8: Paired within-string modality comparison; Table S9: Variance decomposition by description-length band; Table S10: Repeated-string inventory with per-string coefficients of variation; Table S11: Replicate composition—mixed-modality strings and stratum overlap; Table S12: Silent failure of the vision stage—artefact-string prevalence, odds ratio and reliability; Data S1: Frozen audit corpus (meal-level fields); Code S1: repra v0.1.0—reference implementation of the six-test battery and the REPRA acceptance test, with its test suite and a script that reproduces the published figures from the frozen corpus.

## Use of Artificial Intelligence

During the preparation of this manuscript the authors used three general-purpose generative AI assistants—ChatGPT (OpenAI), Claude (Anthropic), and Gemini (Google)—in the releases publicly available to the authors during the first half of 2026. The authors did not record the specific model build identifiers at the time of use and therefore state the period of use rather than assert a version they cannot verify. Their use was confined to two supporting tasks. First, drafting and debugging analytical code: every fragment so produced was read, tested, and verified by the authors against the frozen data extract before use, and the complete analysis code is deposited as Code S1, so that any reader can re-run the analysis and confirm every value reported here independently of how the code was drafted. Second, language editing of text written by the authors: every edited sentence was reviewed and either accepted or rewritten by the authors, who are responsible for its content. No AI system was used to conceive the study, to select or specify the analyses, to compute or generate any reported value, to produce any figure or table, to retrieve, select or format references, to interpret the data, or to write any statement presented as a finding. All statistical results reported here were computed from the frozen data extract by the authors’ own code and cross-checked against the source tables. All 57 references were located by the authors, and each digital object identifier was verified against the registry record of the work it cites; no reference was inserted on the suggestion of an AI system. No AI system is listed as an author, and none met any criterion for authorship: every CRediT role declared above was performed by a named human author. The authors have reviewed and edited the output and take full responsibility for the content of this publication, including its accuracy, its originality, and the integrity of the data and analyses reported.
