# Supplementary material for: Internal Consistency Does Not Validate an LLM-Assisted Nutrition Pipeline: A Reference-Standard-Free Forensic Reliability Audit of 13,073 Free-Text Meal Records
Source: Nutrients. 2026 Sep 30;18(19):3233. doi: 10.3390/nu18193233 (PMC13667244; doi:10.3390/nu18193233)
Supplement: Supplementary file 1 [file nutrients-18-03233-s001.zip › Supplementary_Tables_S1-S12.pdf]

Supplementary Materials

Internal consistency does not validate an LLM-assisted nutrition pipeline: a reference-standard-free forensic reliability audit of 13,073 free-text meal records

Data S1 (frozen audit corpus) and Code S1 (repra v0.1.0) are provided as separate files in the same archive.

Table S1. Terminal-digit distribution by nutrient, with and without exact zeros.

Nutrient	Field	n (total)	Exact zeros (%)	Digits in {0,5}, all values (%)	Entropy, all values (bits)	Chi-square, all values (9 df)	n (zeros excluded)	Digits in {0,5}, zeros excluded (%)	Entropy, zeros excluded (bits)	Chi-square, zeros excluded (9 df)	Distinct values	Maximum entropy (bits)
Energy (kcal)	calorie	13073	0.34	92.31	1.2871	62078	13029	92.29	1.29	61730	762	3.3219
Protein (g)	protein	13073	0.83	46.79	3.013	6672	12965	46.35	3.0187	6509	249	3.3219
Carbohydrate (g)	total_carb	13073	1.12	65.02	2.6126	16808	12927	64.62	2.6246	16291	301	3.3219
Fat (g)	total_fat	13073	1.61	48.57	2.9663	7449	12862	47.72	2.9818	6946	268	3.3219
Fibre (g)	dietary_fiber	13073	5.52	22.93	3.2053	1911	12352	18.43	3.1838	2237	138	3.3219
Sugar (g)	sugar	13073	80	92.34	1.0018	78553	2615	61.72	2.3584	4571	91	3.3219

Table S2. Grid adherence — share of values that are exact multiples of 5, 10 and 25.

Nutrient	Field	n	Multiples of 1 (%)	Multiples of 5 (%)	Multiples of 10 (%)	Multiples of 25 (%)	Multiples of 50 (%)	Distinct values	Distinct values (%)
Energy (kcal)	calorie	13073	99.95	92.3	73.34	29.61	25.01	762	5.83
Protein (g)	protein	13073	96.27	46.09	19.77	9.25	1.96	249	1.9
Carbohydrate (g)	total_carb	13073	98.09	64.84	34.26	11.99	6.35	301	2.3
Fat (g)	total_fat	13073	94.06	47.01	23.93	7.37	1.98	268	2.05
Fibre (g)	dietary_fiber	13073	94.06	22.08	10.38	5.59	5.52	138	1.06
Sugar (g)	sugar	13073	99.37	92.17	82.96	81.17	80	91	0.7

Table S3. ICC sensitivity to the repetition threshold and to the exclusion of artefact strings.

Subset	Strings	Meals	ICC(1,1)	95% CI lower	95% CI upper
All repeated strings (>=3 replicates)	191	944	0.5905	0.4917	0.6788
Strings from a single registrant	31	138	0.8535	0.7086	0.9495
Strings from >=2 registrants	160	806	0.5632	0.4436	0.6539
Text-only (no photograph attached)	85	352	0.7061	0.5557	0.8208
Photograph-accompanied	82	427	0.5258	0.3884	0.6395
Threshold >=5 replicates	61	517	0.5953	0.4696	0.6885
Threshold >=10 replicates	13	215	0.6424	0.4116	0.7729
Undeduplicated (incl. test accounts and re-ingestions)	229	1190	0.5658	0.4807	0.6593
Photograph-accompanied, artefact strings excluded	78	344	0.5593	0.4069	0.7096
Text-only, artefact strings excluded	85	352	0.7061	0.5415	0.8249

Text minus photograph contrast (delta ICC)			0.1803	-0.0164	0.3658

Table S4. Specificity analysis — five simulated non-generative data-producing mechanisms.

Simulated mechanism	Generative	n	Digits in {0,5} (%)	Entropy (bits)	Flagged
Exact table lookup, no rounding	No	13073	20	3.3218	No
Regression model output, rounded to integer	No	13072	19.98	3.3213	No
Human self-report with digit heaping	No	13073	83.5	1.2781	Yes
Composition table displayed rounded to 5 kcal	No	13073	100	1	Yes
Packaged-food database, rounded to 10 kcal	No	13073	100	-0	Yes
OBSERVED - LLM pipeline (energy)	Yes	13029	92.29	1.29	Yes

Table S5. Attenuation bridge — per-participant correlations with fasting glycaemia and noise-corrected estimates.

Exposure	n	r observed	95% CI lower	95% CI upper	p	r corrected (lambda)	Exceeds detection floor
Energy (kcal), per-participant mean	62	-0.143	-0.3792	0.1108	0.2677	-0.1608	No
Protein (g), per-participant mean	62	-0.0951	-0.3368	0.1585	0.4623	-0.107	No
Carbohydrate (g), per-participant mean	62	-0.0946	-0.3364	0.1589	0.4646	-0.1064	No
Fat (g), per-participant mean	62	-0.1318	-0.3694	0.122	0.3072	-0.1483	No
Fibre (g), per-participant mean	62	-0.008	-0.2573	0.2422	0.9506	-0.009	No
Sugar (g), per-participant mean	62	0.0278	-0.2236	0.2756	0.8304	0.0312	No
Detection floor (80% power, alpha=0.05)	62	0.3494				0.3931	

Table S6. Attenuation scenarios, detection thresholds and required sample size.

Scenario	lambda	Detectable observed |r|	Smallest detectable true |r|	Increase in the floor (%)	n needed for a true r of 0.30	Effective sample size
No estimator noise	1	0.3494	0.3494	0	85	62
Per-participant mean (primary)	0.79	0.3494	0.3931	12.5	109	49
Worst case: reliability of 1-2-word strings	0.5668	0.3494	0.4641	32.8	152	35.1
Meal-level analysis (k = 1)	0.5905	0.3494	0.4547	30.1	146	36.6

Table S7. Robustness of the modality contrast across three specifications.

Analysis	ICC, text-only	ICC, photograph	Delta ICC	95% CI lower	95% CI upper	P(text > photograph)	Conclusion
ICC text vs photograph, threshold >=3 replicates	0.7061	0.5258	0.1803	-0.0164	0.3658	0.9635	CI crosses zero
ICC text vs photograph, threshold >=2 replicates	0.6979	0.6122	0.0857	-0.0746	0.2273	0.859	contrast attenuates to 0.086
ICC text vs photograph, artefact strings excluded	0.7061	0.5593	0.1468				sign persists, no CI computed

Table S8. Paired within-string modality comparison.

Quantity	Value	Note
Strings with >=2 replicates of BOTH modalities	30	
Median within-string CV, text replicates	0.1831	
Median within-string CV, photograph replicates	0.2899	
Paired Wilcoxon, W statistic	219.0	
Paired Wilcoxon, p value	0.7922	
Strings where the photograph replicates are more variable	17	of 30
VERDICT	The modality contrast is NOT robust	not sustained as a finding

Table S9. Variance decomposition by description-length band.

Quantity	Value	Unit
MSW (estimator noise, meal level)	15601	kcal^2
SD of estimator noise	124.9	kcal
Noise as % of the corpus median (420 kcal)	29.7	%
True between-string variance	22500	kcal^2
Noise as % of MSW + true between-string variance	40.9	%
Variance of the repeated subset (MSB/MSW design)	37922	kcal^2
Noise as % of the variance of the repeated subset (reported in Section 3.5)	41.1	%
Total variance of the full corpus	51743	kcal^2
SD of the full corpus	227.5	kcal
Noise as % of the total corpus variance	30.2	%
MSW, 1-2-word strings	24634	kcal^2
MSW, 3-5-word strings	10782	kcal^2
MSW, >=6-word strings	1342	kcal^2
Median within-string CV (energy)	0.2254	dimensionless
Strings with CV > 0.20 (%)	54.45	%
Strings with CV = 0, i.e. always the same value (%)	2.09	%
Spearman rho between CV and word count	-0.5931	dimensionless
p of that correlation	1.57e-19	dimensionless
Entropy as % of the maximum (energy)	38.8	%
Noise in the per-participant mean	1243	kcal^2
SD of noise in the per-participant mean	35.3	kcal
Varianza observada entre participantes	5922	kcal^2
Between-participant SD	77	kcal
Meals with energy > 0 (n for T5)	13029	comidas
Meals within +/-10% of the Atwater reconstruction (%)	96.2	%
Delta ICC texto - foto	0.1803	dimensionless

Contrast, 95% CI lower	-0.0164	dimensionless
Contrast, 95% CI upper	0.3658	dimensionless
P(ICC texto > ICC foto)	0.9635	probabilidad
Observation window length	179	dias
Atwater relative error, 25th percentile	-0.0185	proporcion
Atwater relative error, 75th percentile	0.0233	proporcion
Subestudio umbral >=2: cadenas	556	cadenas
Subestudio umbral >=2: comidas	1674	comidas
Subestudio umbral >=2: cadenas solo texto	248	cadenas
Subestudio umbral >=2: cadenas solo foto	249	cadenas
ICC agregado, umbral >=2	0.628	dimensionless
r energia-glucemia corregida, redondeo a 3 dec	0.161	dimensionless
Bootstrap seed	2.02608e+07	entero
Bootstrap replicates, ICC intervals	1000	entero
Bootstrap replicates, modality contrast	2000	entero
Python version	3.11	version
p reported for chi-square below this threshold	0.001	valor p

Table S10. Repeated-string inventory with per-string coefficients of variation.

Normalised string	n	Mean	SD	Minimum	Maximum	Registrants	With photograph (%)	CV	Max/min ratio
comida variada	45	529.13	168.92	275	1050	38	100	0.32	3.82
arroz con pollo	26	474.04	110.14	250	650	20	30.8	0.23	2.6
comida	16	512.81	167.48	270	800	14	100	0.33	2.96
comida analizada	16	403.44	116.94	220	655	13	100	0.29	2.98
huevo	16	290.19	210.86	68	860	13	87.5	0.73	12.65
huevos	14	239.64	95.43	142	470	11	78.6	0.4	3.31
ensalada	14	166.79	154.14	45	525	10	85.7	0.92	11.67
tamal	12	607.5	91.07	485	815	12	50	0.15	1.68
sopa	12	265.92	151.69	38	480	9	100	0.57	12.63
arepa y huevos revueltos	12	336.25	50.43	270	450	9	75	0.15	1.67
cafe negro	11	2.82	1.66	0	5	7	72.7	0.59	
papaya	11	79.27	31.57	43	130	8	63.6	0.4	3.02
cafe con leche	10	119	31.43	50	150	10	50	0.26	3
arepa con huevos revueltos	9	374.44	61.26	300	520	8	77.8	0.16	1.73
agua con limon	9	36.56	99.57	0	302	7	11.1	2.72	
pan y cafe sin azucar	9	80	0	80	80	1	0	0	1
platano asado	8	431	214.98	120	785	7	0	0.5	6.54
huevos y arepa	8	322.62	52.78	260	404	7	100	0.16	1.55
2 huevos cocidos y aromatica	8	155.62	0.52	155	156	1	0	0	1.01
comida no especificada	8	471.88	194.46	230	850	8	100	0.41	3.7
aguacate	8	306.5	167.36	67	575	7	50	0.55	8.58
manzana	8	90.75	47.26	24	190	8	50	0.52	7.92
huevos revueltos	8	273	94.55	140	440	7	87.5	0.35	3.14
huevos revueltos y arepa	7	351.86	50.82	290	425	7	71.4	0.14	1.47
desayuno	7	395.71	254.42	0	800	7	85.7	0.64	

dos panes y un vaso con cafe sin azucar	7	238.57	38.91	160	280	1	0	0.16	1.75
sopa de verduras	7	303.86	310.6	57	780	5	85.7	1.02	13.68
sancocho y cafe	7	452.86	161.94	210	600	1	57.1	0.36	2.86
huevo duro galleta integral y cafe	7	134.29	24.4	120	170	1	0	0.18	1.42
cafe y galletas	7	250.71	116.99	130	490	5	0	0.47	3.77
lentejas	7	297.86	146.06	150	570	6	42.9	0.49	3.8
huevo y arepa	6	249.67	28.72	210	280	6	50	0.12	1.33
limonada	6	176.67	182.94	100	550	4	0	1.04	5.5
lechona	6	630	175.5	400	850	6	0	0.28	2.12
lentejas y arroz	6	377.5	131.29	270	620	4	50	0.35	2.3
manzana verde	6	160	159.22	95	485	5	66.7	1	5.11
lentejas con arroz	6	300.83	79.65	200	390	5	0	0.26	1.95
arepa	6	224.17	108.92	145	430	3	83.3	0.49	2.97
arepa con queso	6	295	52.06	230	380	6	66.7	0.18	1.65
vaso de avena	6	158.33	20.41	150	200	1	0	0.13	1.33
frijoles	6	393.33	286.49	85	875	6	50	0.73	10.29
arepa y cafe	6	192	40.57	145	240	6	16.7	0.21	1.66
sancocho de pollo	6	562.5	228.64	400	1000	5	33.3	0.41	2.5
sandwich	6	456.67	153.58	240	640	6	100	0.34	2.67
huevo duro cafe y galletas integrales	5	254	13.42	240	270	1	0	0.05	1.12
frijoles arroz carne y jugo de pina	5	591	66.37	500	655	1	0	0.11	1.31
avena con leche	5	208	40.87	160	270	4	60	0.2	1.69
huevo duro galleta integral y cafecito	5	180	0	180	180	1	0	0	1
cafe con galletas	5	224	75.03	150	350	5	20	0.34	2.33
dos huevos cocidos	5	208.8	80.67	140	312	3	0	0.39	2.23
cafe	5	2.6	1.34	2	5	5	40	0.52	2.5
desconocido	5	280	271.02	0	550	5	80	0.97	
vaso de avena en leche sin azucar	5	270	0	270	270	1	20	0	1
arroz y papa	5	701	51.28	650	755	5	100	0.07	1.16
huevos pericos y arepa	5	367.4	21.82	350	395	5	20	0.06	1.13
arepa con cafe	5	185.4	54.87	145	280	5	20	0.3	1.93
arepa con huevo	5	315	97.21	225	420	5	20	0.31	1.87
arroz y platano maduro	5	649	72.49	530	700	5	100	0.11	1.32

arroz y platano frito	5	597	91.49	520	750	4	100	0.15	1.44
acema y cafe sin azucar	5	164	42.19	130	210	1	0	0.26	1.62
pollo y arroz	5	485.6	267.43	33	720	3	100	0.55	21.82
huevos pericos arroz cafe	4	317.5	73.2	240	380	1	100	0.23	1.58
2 huevos cocidos y 1 taza de cafe con leche	4	226.25	59.63	170	285	1	100	0.26	1.68
sandwich y banano	4	445	81.85	380	550	4	100	0.18	1.45
sancocho	4	522.5	90.97	450	650	4	25	0.17	1.44
salchipapa	4	651.25	137.98	485	800	3	50	0.21	1.65
dos acemas y un vaso con cafe sin azucar	4	286.25	24.96	260	320	1	0	0.09	1.23
pony malta	4	165	34.16	120	200	4	75	0.21	1.67
omelette	4	323.75	64.73	280	420	3	75	0.2	1.5
pescado y ensalada	4	226.25	63.95	150	300	4	50	0.28	2
omelette con queso y aguacate	4	441.25	38.38	385	470	1	100	0.09	1.22
huevos cocidos	4	265.25	97.69	156	390	4	50	0.37	2.5
mazamorra con leche	4	306.25	42.7	250	350	4	0	0.14	1.4
cereal con leche	4	257.5	45.18	205	315	4	75	0.18	1.54
leche	4	104	31.37	80	150	4	25	0.3	1.88
jugo de maracuya	4	175	125.83	80	360	2	25	0.72	4.5
huevos revueltos y cafe negro	4	195	33.17	160	240	1	100	0.17	1.5
huevos revueltos con arroz	4	357.5	109.05	250	500	4	75	0.3	2
sudado de pollo con arroz y ensalada	4	415	10	400	420	2	0	0.02	1.05
2 huevos cocidos aromatica y queso	4	268.25	24.5	256	305	1	0	0.09	1.19
arepa y cafe con leche	4	317.5	73.65	230	400	4	25	0.23	1.74
3 huevos cocidos y 1 taza de cafe con leche	4	279.75	25.7	260	314	1	100	0.09	1.21
arepa y huevo cocido	4	230	23.09	210	250	3	0	0.1	1.19
cafe y pan	4	141.5	16.42	120	160	2	75	0.12	1.33
cafe con leche y arepa	4	261.25	51.05	200	320	4	50	0.2	1.6
arepa y huevo	4	295	94.69	210	430	4	75	0.32	2.05
arroz y lentejas	4	348.75	57.5	320	435	3	0	0.16	1.36
agua	4	83.75	167.5	0	335	4	50	2	

arroz y frijoles	4	403.75	246.49	130	705	4	75	0.61	5.42
arroz con pollo y cafe	4	422.5	88.84	290	480	1	0	0.21	1.66
arepa y cafe sin azucar	4	126.5	9	122	140	1	0	0.07	1.15
carne asada arroz y ensalada	4	312.5	114.64	195	470	3	50	0.37	2.41
arroz con pollo y limonada	4	562.5	90.32	430	620	3	25	0.16	1.44
arroz con leche	4	361.25	132.94	245	500	4	0	0.37	2.04
batido	4	177.5	18.93	150	190	4	75	0.11	1.27
carne asada y ensalada	4	282.5	50.41	210	325	4	50	0.18	1.55
carne y ensalada	4	238.25	27.97	210	275	3	50	0.12	1.31
arepa con queso y cafe	4	250.5	13.2	232	260	4	0	0.05	1.12
pechuga asada y ensalada	3	199	16.82	180	212	3	33.3	0.08	1.18
pollo asado	3	340	40	300	380	3	33.3	0.12	1.27
pescado y arroz	3	283.33	141.54	120	370	2	33.3	0.5	3.08
pescado y arepa	3	440	144.22	320	600	2	100	0.33	1.88
pescado sudado	3	331.67	122.71	190	405	3	66.7	0.37	2.13
cereal	3	195	25.98	180	225	1	0	0.13	1.25
pastel	3	403.33	15.28	390	420	2	66.7	0.04	1.08
pandebono y cafe	3	260	26.46	240	290	3	0	0.1	1.21
arepas y huevos revueltos	3	518.33	171.2	360	700	2	100	0.33	1.94
pollo asado y arroz	3	351.67	71.47	290	430	3	33.3	0.2	1.48
arroz blanco	3	391.67	137.69	250	525	3	100	0.35	2.1
muslo de pollo cocido o asado y ensalada de zanahoria rallada y repollo	3	266.67	2.89	265	270	1	100	0.01	1.02
arroz blanco con huevo revuelto	3	323	79.04	234	385	3	100	0.24	1.65
mazamorra	3	266.67	49.33	210	300	2	0	0.18	1.43
arroz blanco y huevos revueltos	3	405	43.3	355	430	2	100	0.11	1.21
mandarina	3	240	257.23	88	537	3	0	1.07	6.1
arroz	3	363.33	253.59	195	655	3	66.7	0.7	3.36
arepa huevos revueltos y cafe con leche	3	456.67	28.87	440	490	2	100	0.06	1.11
pollo sudado con papa y ensalada	3	378.33	70.06	300	435	3	0	0.19	1.45
arepa queso y chocolate	3	413.33	57.74	380	480	2	100	0.14	1.26
vaso de	3	125.33	27.3	96	150	3	33.3	0.22	1.56

leche									
2 huevos revueltos	3	261.67	92.51	155	320	2	33.3	0.35	2.06
2 huevos y aromatica	3	208	90.07	156	312	1	0	0.43	2
tinto y pandebono	3	295	13.23	280	305	2	0	0.04	1.09
te	3	1.67	2.89	0	5	3	0	1.73	
sudado de pollo y ensalada	3	293.33	30.55	260	320	3	0	0.1	1.23
sudado de pollo y arroz	3	463.33	63.51	390	500	3	0	0.14	1.28
sudado de pollo con arroz	3	583.33	94.52	510	690	2	0	0.16	1.35
sopa o crema	3	196.67	40.41	150	220	3	100	0.21	1.47
sopa de verduras pechuga asada y ensalada	3	268.33	40.72	240	315	1	100	0.15	1.31
ajiaco	3	520	60.83	450	560	3	66.7	0.12	1.24
arepa asada	3	248.33	174.67	145	450	3	66.7	0.7	3.1
sandwich y cafe sin azucar	3	283.33	15.28	270	300	1	0	0.05	1.11
arepa con perico y cafe con leche	3	431.67	14.43	415	440	2	100	0.03	1.06
sandwich con queso y banano	3	506.67	44.81	455	535	3	100	0.09	1.18
sandwich chocolate y mango	3	510	43.59	480	560	3	0	0.09	1.17
sandia	3	60	15	45	75	3	100	0.25	1.67
arepa de maiz	3	256.67	168.62	140	450	3	100	0.66	3.21
arepa huevo cocido y avena	3	403.33	15.28	390	420	1	0	0.04	1.08
arepa huevos revueltos cafe con leche	3	536.67	88.08	465	635	2	100	0.16	1.37
rueda de pan y cafe sin azucar	3	98	29.46	80	132	1	0	0.3	1.65
maiz pira	3	109	46.51	62	155	2	33.3	0.43	2.5
lentejas arroz y carne	3	472.67	150.87	310	608	3	0	0.32	1.96
lentejas y ensalada	3	376.67	92.92	300	480	3	66.7	0.25	1.6
ensalada con atun	3	320	36.06	280	350	2	0	0.11	1.25
huevo frito	3	116.67	46.19	90	170	3	33.3	0.4	1.89
cafe con leche y pan integral	3	170	30	140	200	3	0	0.18	1.43
cafe con pan	3	185	47.7	130	215	2	66.7	0.26	1.65
huevo duro cafe galletas integrales	3	213.33	11.55	200	220	1	0	0.05	1.1
cafe negro	3	2	0	2	2	2	33.3	0	1

sin azucar									
higado encebollado	3	363.33	118.78	285	500	3	0	0.33	1.75
frijoles arroz y ensalada	3	318.33	97.77	210	400	2	0	0.31	1.9
frijoles arroz ensalada	3	291.67	82.21	230	385	2	0	0.28	1.67
cafe y bunuelo	3	320	70	250	390	3	33.3	0.22	1.56
espagueti	3	273.33	46.19	220	300	2	0	0.17	1.36
caldo	3	16.67	7.64	10	25	3	33.3	0.46	2.5
arroz con pollo y ensalada	3	560	87.18	460	620	2	0	0.16	1.35
caldo de costilla	3	220	124.9	80	320	3	0	0.57	4
dos huevos fritos y una taza de cafe negro	3	180	20	160	200	3	100	0.11	1.25
caldo de pescado	3	203.33	5.77	200	210	3	66.7	0.03	1.05
caldo de pollo	3	85	27.84	55	110	3	0	0.33	2
caldo de pollo con papas	3	333.33	75.72	280	420	3	66.7	0.23	1.5
carne	3	530	350.43	190	890	3	33.3	0.66	4.68
carne asada	3	458.33	382.5	235	900	3	0	0.83	3.83
comida estimada	3	423.33	230.07	190	650	3	100	0.54	3.42
comida del dia	3	471.67	156.87	355	650	3	100	0.33	1.83
comida con proteina y vegetales	3	441.67	59.65	375	490	3	100	0.14	1.31
huevo y aguacate	3	433.33	106.93	340	550	2	100	0.25	1.62
bunuelo	3	268.33	60.48	200	315	3	66.7	0.23	1.58
huevo y arroz	3	320	285.83	150	650	2	100	0.89	4.33
huevo y pan integral	3	230	52.92	170	270	2	66.7	0.23	1.59
lentejas ensalada y carne asada	3	498.33	20.21	475	510	1	100	0.04	1.07
combinacion de alimentos	3	428.33	84.61	335	500	3	100	0.2	1.49
lentejas arroz ensalada	3	296.67	50.33	250	350	3	33.3	0.17	1.4
arroz y carne asada	3	523.33	240.07	370	800	3	100	0.46	2.16
jugo de mora	3	249.33	189.37	140	468	2	0	0.76	3.34
arroz y platano	3	620	60	560	680	3	100	0.1	1.21
arroz y platano maduro frito	3	625	92.6	535	720	3	100	0.15	1.35
arroz y pollo asado	3	390	157.4	215	520	3	0	0.4	2.42
huevos revueltos y cafe con leche	3	293.33	53.93	255	355	3	100	0.18	1.39
atun	3	370	459.02	100	900	3	0	1.24	9
huevos	3	289	19.05	267	300	1	66.7	0.07	1.12

revueltos con zanahoria espinacas y media arepa									
huevos revueltos con vegetales y arepa	3	343.33	9.07	333	350	2	100	0.03	1.05
huevos revueltos con vegetales	3	218.33	55.75	155	260	2	100	0.26	1.68
huevos revueltos con tomate y cebolla	3	200	40	160	240	3	66.7	0.2	1.5
atun y aguacate	3	310	121.24	180	420	3	66.7	0.39	2.33
avena	3	198.33	75.22	150	285	3	66.7	0.38	1.9
avena con galletas integrales	3	308.33	29.3	275	330	2	66.7	0.1	1.2
banano	3	186.67	141.45	105	350	3	66.7	0.76	3.33
huevos fritos y arepa	3	316.67	28.87	300	350	3	100	0.09	1.17
huevos cocidos y arepa	3	338.33	52.04	280	380	3	100	0.15	1.36
bandeja paisa	3	1120	158.75	1000	1300	3	0	0.14	1.3
yogur con fresas	3	127.33	34.15	90	157	1	0	0.27	1.74

Table S11. Replicate composition — mixed-modality strings and stratum overlap.

Quantity	Value
Repeated strings (>=3) in the cleaned analytical set	191
Of these, mixed (replicates with and without a photograph)	86
Of these, text-only	57
Of these, photograph-only	48
Repeated strings that are mixed (%)	45
Repeated strings in the UNcleaned set (window, incl. test accounts and re-ingestions)	229
Of those 229, mixed	101
Strings in the 'text replicates' ICC stratum (>=3 replicates without a photograph)	85
Of these, strings that are mixed at source	28
Strings in the 'photograph replicates' ICC stratum (>=3 replicates with a photograph)	82
Of these, strings that are mixed at source	34

Table S12. Silent failure of the vision stage — artefact-string prevalence, odds ratio and reliability.

Quantity	Value	Unit
Records, photograph-accompanied stratum	6320	records
Records, text-only stratum	6753	records
Artefact strings, photograph stratum	91	records
Artefact strings, text-only stratum	1	records
Artefact-string prevalence, photograph stratum	1.44	%
Artefact-string prevalence, text-only stratum	0.01	%
Odds ratio, photograph vs text-only	98.6	ratio
Share of all artefact strings accompanied by a	98.9	%

photograph		
Artefact strings entering the test-retest substudy	5	strings
Meals contributed by artefact strings	90	of 944 (9.5%)
ICC(1,1) energy, artefact strings, photograph stratum (89 meals)	0.0911	ICC
ICC(1,1) energy, artefact strings, both strata (90 meals)	0.148	ICC
ICC(1,1) energy, pooled corpus, all strings (191 strings, 944 meals)	0.5905	ICC
ICC(1,1) energy, photograph stratum, all strings (82, 427)	0.5258	ICC
ICC(1,1) energy, photograph stratum, artefact strings excluded (77, 338)	0.572	ICC
ICC(1,1) energy, pooled, artefact strings excluded (186, 854)	0.6088	ICC
Within-string CV, 'comida variada' (45 submissions, 275-1050 kcal)	0.319	CV
Within-string CV, 'desconocido' (5 submissions, 0-550 kcal)	0.968	CV
